# Gut microbiota-host lipid crosstalk in Alzheimer’s disease: implications for disease progression and therapeutics

**DOI:** 10.1186/s13024-024-00720-0

**Published:** 2024-04-16

**Authors:** Ya-Xi Luo, Ling-Ling Yang, Xiu-Qing Yao

**Affiliations:** 1 Department of Rehabilitation, The Second Affiliated Hospital of Chongqing Medical University, Chongqing, China; 2 Chongqing Municipality Clinical Research Center for Geriatric Medicine, Chongqing, China; 3 Department of Rehabilitation Therapy, Chongqing Medical University, Chongqing, China

**Keywords:** Gut microbiota, Lipid metabolism, Alzheimer’s disease, Cholesterol, SCFAs, LPS, APOE, Neuroinflammation, Probiotics, Lifestyle, Exercise

## Abstract

Trillions of intestinal bacteria in the human body undergo dynamic transformations in response to physiological and pathological changes. Alterations in their composition and metabolites collectively contribute to the progression of Alzheimer’s disease. The role of gut microbiota in Alzheimer’s disease is diverse and complex, evidence suggests lipid metabolism may be one of the potential pathways. However, the mechanisms that gut microbiota mediate lipid metabolism in Alzheimer’s disease pathology remain unclear, necessitating further investigation for clarification. This review highlights the current understanding of how gut microbiota disrupts lipid metabolism and discusses the implications of these discoveries in guiding strategies for the prevention or treatment of Alzheimer’s disease based on existing data.

**Supplementary Information:**

The online version contains supplementary material available at 10.1186/s13024-024-00720-0.

## Background

Alzheimer’s disease (AD), constituting 60-70% of dementia cases, is the most prevalent cause of dementia, with global estimates surpassing 50 million patients, thereby imposing a substantial societal and familial burden [1]. Clinically, AD manifests with progressive memory loss, cognitive impairment, and behavioral changes. Neuropathologically, β-amyloid (Aβ) deposits extracellularly, forming neuroinflammatory plaques, while intracellularly, excessive phosphorylation of tau leading to the formation of neurofibrillary tangles (NFTs), accompanied by synaptic loss and neurodegeneration [2–4]. Moreover, abnormalities in lipid metabolism have been identified as the third pathological feature of AD, associated with disease onset and progression [5]. Lipids, constituting 50% of the brain’s weight, encompassing fatty acids, cholesterol, phospholipids, and sphingolipids, play a pivotal role in brain function [6]. For instance, fatty acid oxidation contributes 20% of the brain’s energy supply [7], and cholesterol and sphingolipids are major components of lipid rafts [8, 9], playing crucial roles in neurotransmitter transmission, signal transduction, and neural synaptic plasticity. Alterations in lipid homeostasis manifest in the early stages of AD [10], with studies indicating abnormal lipid deposition in the brains of AD patients and 3×Tg AD mice, signifying disrupted lipid metabolism [11–14]. Concurrently, a systematic review also encapsulated the prevalent occurrence of lipid dysregulation in AD mouse models such as 5×FAD, APP/PS1, among others [15]. Metabolic analysis of serum, plasma, and cerebrospinal fluid from AD patients reveals the dysregulation of lipid metabolism is closely linked to cognitive decline and neuronal dysfunction [16, 17].

The gut microbiota, populating the human gastrointestinal tract, consists of approximately 100 trillion bacteria, archaea, and eukaryotes, collectively encoding over 3 million genes and generating a diverse array of metabolites [18]. Through the bidirectional “gut-brain axis” [19, 20], the gut microbiota actively modulates host metabolic processes [21–24]. During pathological conditions, dynamic alterations in the gut microbiota have been observed in tandem with the progression of the host’s disease [25–27]. 16S rRNA gene sequencing of gut microbiota in AD patients reveals a reduction in both abundance and diversity [28–30], such as, the significant correlation between the increased abundance of *Enterobacteriaceae* and the severity and progression of AD has been observed [30]. The alterations in the composition of the gut microbiota have also been associated with Aβ and tau pathological biomarkers [31]. Our study establishes *Helicobacter pylori* (H. pylori) infection as a risk factor for AD [32, 33], 5×FAD mice [34], and Tauopathy mouse model [25] has been provided. Chandra S and colleagues have extensively summarized these findings [35]. Mezö et al. found that Germ-free (GF) 5×FAD mice enhance the uptake of Aβ deposits by hippocampal microglia, mitigating plaque burden and neuronal loss, and improving memory function [36]. Antibiotic treatment in APP/PS1 mice also led to a reduction in Aβ deposition [37]. Harach et al. reached the same conclusion in GF APP/PS1 mice [38], providing compelling evidence for the role of gut microbiota in AD. Transplanting gut microbiota from AD mice into wild-type C57BL/6 mice results in impaired memory function and neurogenesis [39]. Conversely, fecal microbiota transplantation from healthy mice effectively mitigates Aβ plaque deposition and neurofibrillary tangle formation in AD mice, thereby ameliorating cognitive impairment [40]. These findings underscore the complex involvement of gut microbiota in AD pathology and suggest its potential as a therapeutic avenue for AD.

Emerging evidence suggests a significant interaction between gut microbiota and lipid metabolism in AD [41]. Such as correlations were observed between gut microbiota and fatty acids as well as glycerophospholipids in APP/PS1 mice [42]. A recent multi-omics study has unveiled the intricate connection between gut microbiota and host glycerophospholipid metabolism, as well as neuroinflammation in APP/PS1 mice [43, 44]. Additionally, gut microbiota from 3×Tg mice induced increased pro-inflammatory signal transduction through polyunsaturated fatty acid metabolism [45]. Multi-strain probiotic formulation resulted in reduced total cholesterol levels, improved lipid metabolism, and enhanced cognitive function [46]. These findings present compelling evidence for the involvement of gut microbiota in the lipid metabolism of AD.

Given the pivotal roles of lipid metabolism and gut microbiota in AD, this review systematically explores the dysregulation of lipid metabolism in the context of AD. Subsequently, it emphasizes the intricate interplay between gut microbiota and lipid metabolism during AD pathology, with a specific focus on gut microbiota metabolites, key genes, and molecular mechanisms. Lastly, the paper provides a comprehensive summary and analysis of current research pertaining to strategies aimed at modulating gut microbiota and lipid metabolism, to improve the pathological progression of AD.

## Lipids dysregulation in Alzheimer’s disease

Decades of research have unveiled the involvement of lipid metabolism in the pathological processes of AD. This section primarily elucidates the changes and roles of lipids dysregulation in AD pathology from the perspectives of fatty acids, cholesterol, phospholipids, and sphingolipids.

### Fatty acids

Fatty acids, characterized by long hydrocarbon chains, are categorized into saturated fatty acids (SFAs) and unsaturated fatty acids (UFA), which encompass both monounsaturated and polyunsaturated fatty acids (PUFAs) [47]. ω-3 polyunsaturated fatty acids (ω-3 PUFAs), including docosahexaenoic acid (DHA) and eicosapentaenoic acid (EPA), as well as ω-6 polyunsaturated fatty acids (ω-6 PUFAs) like arachidonic acid (AA), have been identified to play a significant role in AD [48]. Metabolomic analysis of post-mortem samples from the Baltimore Longitudinal Study of Aging (BLSA) cohort revealed the strongest correlation between PUFAs and AD [49]. Current research suggests that ω-3 PUFAs, such as EPA and DHA, exert anti-inflammatory effects, mitigating Aβ deposition and improving cognition in AD mice [50]. DHA as the most abundant PUFAs in the brain, reduces amyloid production and inhibits the aggregation and formation of amyloidogenic fibrils by decreasing the activity of γ- and β-secretase enzymes, and its levels are significantly reduced in the brains and plasma of AD patients [49, 51, 52]. In contrast to ω-3 PUFAs, ω-6 PUFAs are recognized as pro-inflammatory precursors, contributing to the synthesis of leukotrienes, prostaglandins, and thromboxane [53]. ω-6 PUFAs activate GPR40 activation, which is implicated in neuronal degeneration and death; thus, the excessive intake of ω-6 PUFAs is considered a risk factor for AD [54]. AA, a plentiful ω-6 PUFA found in the gray matter of the brain, exhibits heightened levels in individuals with AD, thereby fostering the generation and accumulation of Aβ through a series of inflammatory cascades. Despite the prevailing perception of ω-3 PUFAs as protective elements for AD patients, it is crucial to recognize that both ω-3 and ω-6 PUFAs are integral to brain physiology. Therefore, it is imperative to uphold a judicious balance in the intake of ω-6/ω-3 PUFAs for optimal individual well-being [55].

SFAs are generally considered to increase the risk of AD [56]. Such as palmitic acid (PA), the most common SFAs in the brain, has been found to have elevated levels in the temporal and frontal cortices of AD patients [57, 58] PA induces SIRT1 dysfunction and activates the NF-κB pathway [59]. Through SIRT1 inhibition, PA indirectly upregulates SREBP1 transcription and expression, leading to β-site amyloid precursor protein-cleaving enzyme 1(BACE1) promoter transactivation. This cascade results in increased BACE1 expression, heightened enzymatic activity, and elevated amyloid-beta generation. Additionally, PA induces tau hyperphosphorylation and neuroinflammation [60].

### Cholesterol

In the presence of the blood-brain barrier, cerebral cholesterol primarily originates from endogenous synthesis. In brief, astrocyte-synthesized cholesterol, coupled with apolipoprotein E and J, is secreted through ATP-binding cassette transporters, followed by neuronal uptake. Excessive intracellular free cholesterol is enzymatically converted into cholesterol ester (CE) by cholesterol acyltransferase, leading to intracellular accumulation or plasma membrane efflux [5]. Increased brain cholesterol concentrations are regarded as a risk factor for AD due to the robust association with synaptic dysfunction and impaired neurotransmission, as evidenced in brain tissue from both AD patients and mouse models [61]. Post-mortem examinations of AD patients revealed an association between hypercholesterolemia and increased accumulation of Aβ in the brain [62]. The observed association could stem from blood-brain barrier impairment, resulting in an augmented cholesterol influx into the brain and exacerbating the cerebral cholesterol burden. Subsequently, this mechanism accelerates Aβ aggregation within neurons and synaptic loss, thereby aggravating the pathological progression of AD [63].

A cholesterol-binding domain has been reported within the transmembrane domain of amyloid precursor protein (APP), suggesting a direct interaction between cholesterol and APP. Cholesterol upregulates the activity of γ-secretase, promoting the colocalization of APP with γ and β-secretases, thereby stimulating the generation of Aβ [64, 65]. A reduction in cholesterol may enhance non-pathogenic cleavage by α-secretase, consequently reducing Aβ production [66]. In addition, CE can bind to the cholesterol-binding domain on APP, affecting APP processing and Aβ production. In contrast to the Aβ regulation, CE regulate the phosphorylation of tau in neurons through a proteinase-pTau axis. The reduction in CE generation leads to a decrease in the expression levels of phosphorylated tau at multiple sites [67].

### Phospholipid

Phospholipids form the lipid bilayer of cell membranes, acting as a safeguarding barrier for cellular and subcellular structures. They also participate in maintaining homeostasis, managing immune responses, oxidative stress and neuroinflammation in the brain. The main brain phospholipids are phosphatidylcholine (PC) and phosphatidylethanolamine (PE) [68]. There have been reports that the lyso-PC to PC ratio decreases, and water-soluble PC metabolites increase in individuals with AD [69]. Indeed, increased levels of glycerophosphocholine and decreased levels of lysoPC(18:1(11Z)), PC (16:0/16:0) and phosphatidylcholine were observed in the brains of APP/PS1 transgenic mice [43]. This suggests that there is more hydrolysis of phospholipids during the course of AD. The degradation of PC and PE is considered as a significant metabolic anomaly in AD, with the decreased concentrations being closely associated with the severity of amyloid protein and neurofibrillary pathology [70–72]. Moreover, there is a substantial 70% decrease in ethanolamine plasmalogens (PlsEtns) in AD patients, which exert neuroprotective effects by activating G-protein coupled receptors (GPCRs), increasing AKT and ERK pathway phosphorylation, preventing neuronal death, reducing γ-secretase activity, and decreasing Aβ production [73, 74]. Additionally, mice deficient in PlsEtns have increased activity of the tau phosphorylating kinase glycogen synthase kinase 3 beta (GSK3β), possibly leading to excessive tau phosphorylation [75].

Another noteworthy phospholipid is Phosphatidylserine (PtdSer), constituting 13–15% of phospholipids in the human cerebral cortex [76]. PtdSer serves as an indispensable participant in signal transduction, asymmetrically distributed on the leaflet of the lipid bilayer [77]. PtdSer on the inner leaflet of the membrane facilitates the activation of signaling proteins and receptors crucial for neuronal survival, differentiation, and synaptic neurotransmission [78]. Externalized phosphatidylserine (ePtdSer) serves as one of the ligands for The Triggering Receptor Expressed on Myeloid Cells 2 (TREM2) [79]. ePtdSer exposed on synaptic surfaces acts as an “eat-me” signal, facilitating microglia-mediated synapse pruning [80] and phagocytic activity [81]. In Nondemented individuals with AD neuropathology (NDAN), microglia induced by ePtdSer-TREM2 exhibit enhanced efficiency in clearing damaged synapses, which may underlie the synaptic structural and functional integrity in NDAN individuals, thus preventing cognitive impairment [82]. Under oxidative stress conditions of AD, the asymmetry of PtdSer is disrupted, evidenced by increased exposure of PtdSer in the outer leaflets of the frontal cortex in individuals with Mild Cognitive Impairment (MCI) and AD, initiating early apoptosis and ultimately leading to increased neuronal damage [77]. Another study suggests that in the early stages of AD, the release of ePtdSer serves as an “eat-me” signal, prompting microglia to preferentially eliminate ePtdSer^+^ damaged synapses through TREM2, thereby maintaining neural and synaptic homeostasis. However, microglia expressing dysfunctional R47H TREM2 fail to phagocytose ePtdSer^+^ synapses, resulting in increased apoptotic-like synaptic burden in the hippocampus [83].

### Sphingolipids

Sphingolipids, which are essential components of neuronal cell membranes, include bioactive lipids such as ceramide (Cer), sphingosine-1-phosphate (S1P) and sphingosine, and are involved in the regulation of neuronal stress, proliferation, differentiation and maturation [84]. Metabolomic analysis of brain and blood in preclinical and prodromal stages of AD shows a correlation between decreased sphingolipid levels and the severity of AD pathology [71, 85, 86]. Blood sphingolipid concentrations correlate with cerebrospinal fluid Aβ levels, brain atrophy and cognitive decline, suggesting the potential of sphingolipids as early biomarkers of AD [71].

Ceramides which increased in the brain tissue of AD patients [87] contribute to the stability of BACE1 and facilitate the excessive generation of Aβ [88]. Neurons accelerate Aβ aggregation by releasing extracellular vesicles rich in ceramides, while inhibition or silencing of neutral sphingomyelinase-2 (nSMase2), which is responsible for vesicle secretion, has been shown to improve Aβ plaque formation, slow the pathological progression of AD and improve cognitive function [89]. In addition, S1P, recognized for its neuroprotective effects, exhibits decreased levels in the brain tissues of both AD patients and 5×FAD mice, potentially accelerating neuronal degeneration [87, 89].

## Gut microbial metabolites regulate AD lipids and pathology

**Fig. 1:**
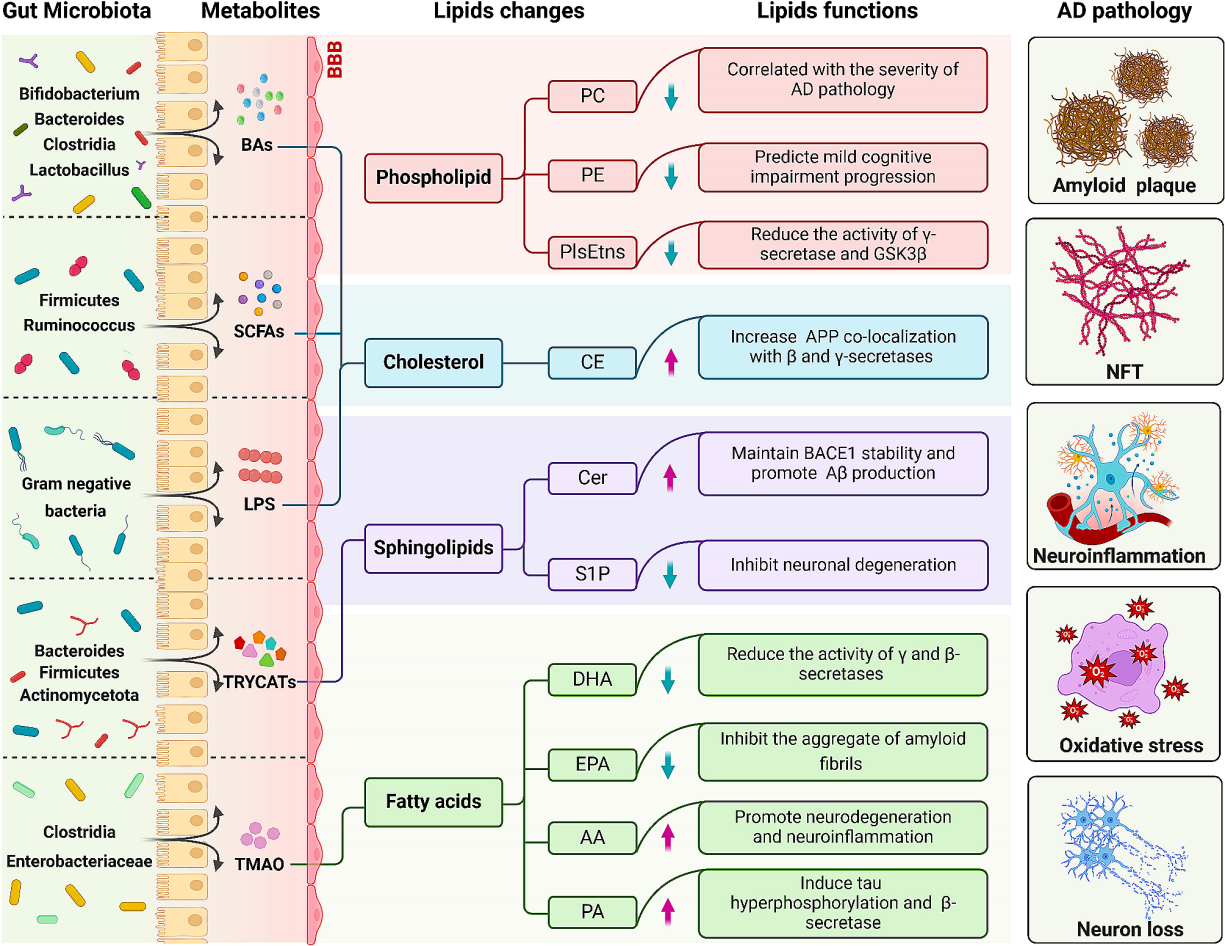
The potential association between gut microbiota and their metabolites with lipid dysregulation in AD. Throughout the progression of Alzheimer’s disease (AD), there is a reduction in phosphatidylcholine (PC) and phosphatidylethanolamine (PE). PC exhibits a negative correlation with the severity of AD pathology, while PE serves as a prognostic indicator for patients with mild cognitive impairment. Plasmalogens (PlsEtns) mitigate tau phosphorylation and experience downregulation in AD. Cholesterol, a pivotal lipid in AD, notably increases in the brain, accompanied by a significant elevation in cholesterol esters (CE). Cholesterol and CE play pivotal roles in AD, contributing to Aβ pathology, tau hyperphosphorylation, and neuroinflammation. Gut microbiota metabolites such as BAs, SCFAs, and LPS interact with cholesterol, thereby modulating AD pathology. Ceramide (Cer) levels escalate in AD, stabilizing BACE1 and fostering Aβ production, whereas sphingosine-1-phosphate (S1P) exhibits neuroprotective effects, and its decrease facilitates neurodegeneration. Tryptophan metabolites (TRYCATs) are intricately associated with sphingolipids through the AhR receptor. Fatty acids like docosahexaenoic acid (DHA) and eicosapentaenoic acid (EPA) decrease in AD, playing a role in inhibiting Aβ generation. Arachidonic acid (AA) is a prominent participant in neuroinflammation, instigating neuronal degeneration. Palmitic acid (PA), a representative saturated fatty acid, fosters AD through heightened β-secretase activity and tau hyperphosphorylation. Trimethylamine N-oxide (TMAO) exacerbates AD progression by promoting fatty acid oxidation and oxidative stress

Gut microbiota generate diverse metabolites, including short-chain fatty acids, bile acids, lipopolysaccharides, trimethylamine, tryptophan metabolites, and more. These metabolites serve as signaling molecules and substrates within the host, thereby regulating host physiological functions [90]. Fluctuations in gut microbiota coincide with alterations in metabolite levels throughout the progression of AD. Several studies have elucidated the regulatory role of bacterial metabolites in lipid metabolism, shedding light on their involvement in the progression of AD pathology (Fig. 1). This section provides a comprehensive summary of studies in the literature investigating the impact of bacterial metabolites on lipid metabolism and pathological progression in AD.

### Short chain fatty acids

Gut microbiota enzymatically degrades carbohydrates and plant polysaccharides to produce SCFAs, particularly acetate, propionic acid, and butyrate, which collectively constitute about 95% of the total SCFAs in the human body. *Bacteroidetes* and *Firmicutes* preferentially generate propionate, *Firmicutes* (*Eubacterium*, *Anaerostipes*, *Roseburia*, and *Faecalibacterium prausnitzii*) are well-defined as the predominant producers of butyrate. *Akkermansia muciniphila* has the unique ability to utilize intestinal mucin as its sole carbon and nitrogen source for proliferation, with propionate as a central metabolite. These SCFAs have the capacity to traverse the blood-brain barrier or the gut-brain axis, exerting regulatory effects on neurotransmitter synthesis, mitochondrial function, lipid metabolism, and gene expression [44].

Targeted metabolomics analysis of feces from AD patients indicates a reduction in the levels of SCFAs-producing phyla such as *Firmicutes*, *Clostridia*, and *Ruminococcus*, resulting in decreased SCFAs levels [91]. SCFAs play a pivotal role in AD pathology through Ligands for G protein-coupled receptors (GPRs) signaling [92]. Specifically, SCFAs bind to GPR43 and GPR109A receptors, modulate cholesterol and lipid metabolism, activate MAPK pathway, and suppress NF-κB pathway [93]. In addition, studies have explored the functions and roles of individual SCFA in AD, such as, acetate up-regulates GPR41 expression, inhibits the ERK/JNK/NF-κB pathway, alleviates neuroinflammation, and improves cognition in APP/PS1 mice [94]. Additionally, propionic acid binds to GPR41 in brain endothelial cells, hinders low-density lipoprotein receptor-related protein 1(LRP-1) expression through a CD14-dependent mechanism, and shields the blood-brain barrier from oxidative stress via NRF2 signaling [95]. Furthermore, epigenetics represents one of the mechanisms by which SCFAs impact AD. Butyrate, acting as a histone deacetylase inhibitor, has demonstrated the ability to elevate the expression of genes associated with

 learning, restore histone acetylation, and markedly enhance learning and memory ability in AD mice [96], acetate also enhances cognition by facilitating histone H3K18 acetylation [97].

Furthermore, Colombo et al. observed a significant reduction in Aβ plaque burden in the brains of GF APP/PS1 mice compared to specific pathogen-free (SPF) APP/PS1 mice, with improved cognitive performance in spatial memory tasks. Subsequent investigations revealed that the increased concentration of SCFAs in the plasma of SPF mice was the primary cause of this outcome, contrary to prior findings; supplementation of SCFAs simulated the role of the microbiota and increased Aβ plaque burden [98]. Erny and colleagues also demonstrated that acetate treatment exacerbated Aβ plaque deposition and neuroinflammation in GF 5×FAD mice [99]. In addition, recent investigation demonstrated that SCFAs act as mediators in the neuroinflammation-neurodegeneration axis, intensifying reactivity in neural glial cells and exacerbating p-tau pathology in TE4 P301S tau transgenic mice [100]. Nevertheless, the experimental findings reported by Zhou et al. indicate that dietary supplementation with high acetate and butyrate (HAMSAB) may attenuate cognitive decline in 5×FAD mice [101], highlighting a potential protective role of endogenous SCFA. These findings suggest the paradoxical roles and functions of SCFAs in AD under different circumstances. Further studies are essential to gain a deeper understanding of the effects of SCFAs on AD. While the role of SCFAs remains controversial, their significance as key molecular mediators in the gut-brain axis of AD is undeniable. Modulation of SCFAs and their related pathways may represent promising therapeutic targets in the early peripheral circulation of AD.

### Bile acids

Bile acids (BAs) can be further subdivided into primary Bas and secondary Bas; the primary Bas consist of cholic acid (CA) and chenodeoxycholic acid (CDCA) [102, 103]. The bile salt hydrolase, which are produced in the intestine by *Bacteroides*, *lostridium cluster VIA, Lactobacillus*, and *Bifidobacterium*, transform primary bile acids into secondary bile acids, which include deoxycholic acid (DCA), lithocholic acid (LCA), and cholic ursodeoxycholic acid (UDCA) [102, 104]. Primary bile acids are responsible for lipid absorption and maintaining cholesterol homeostasis, as they can penetrate the blood-brain barrier (BBB) and bind to nuclear receptors to regulate brain physiological functions. A multicenter metabolomic study involving 1464 participants found that in the serum metabolome of AD patients, DCA and its conjugated forms with glycine and taurine increased, and deoxycholic acid was associated with decreased cognitive abilities [104]. Another metabolomic analysis revealed that the alternative synthesis pathway of bile acids is more active in individuals with AD, and increased serum concentrations of cytotoxic bile acids [103, 105], indicating that dysregulation of bile acid metabolism caused by gut microbiota imbalance is involved in the pathological process of AD.

In addition, some BAs have been reported to exhibit neuroprotective effects. A recent systematic review summarized that UDCA reduces the levels of ROS, tumor necrosis factor alpha (TNFα), and interleukin-1 beta (IL-1β), exerting anti-apoptotic, oxidative stress and inflammatory effects in AD [106]. In addition, tauroursodeoxycholic acid (TUDCA) reduces Aβ deposition, inhibits the progression of amyloid pathology and suppresses GSK3β activity, thereby reducing tau hyperphosphorylation and microglial cell activation [107]. Further research has confirmed that TUDCA can bind to the G protein-coupled bile acid receptor 1/Takeda G protein-coupled receptor 5 (GPBAR1/TGR5) in microglial cells, increasing cAMP levels, inducing an anti-inflammatory phenotype of microglial cells, and attenuating inflammatory responses [108]. These findings indicate the potential therapeutic effects of TUDCA in AD [109, 110].

### Lipopolysaccharides

Lipopolysaccharides (LPS), endotoxins produced by intestinal gram-negative bacteria such as *Escherichia coli*, *Bacteroides fragilis*, and *Salmonella enterica*, were found to coexist with *E. coli* fragments in amyloid plaques in postmortem brain tissues of AD patients [111]. Furthermore, the blood levels of LPS were significantly increased in AD patients, and the presence of LPS was also detected in the neocortex and hippocampus of AD patients at levels more than seven times higher than control subjects [112]. These findings indicate that LPS is widely present in the brains of AD patients and may represent a risk factor for cognitive impairment and the progression of AD [113].

The role of LPS in the pathophysiology of AD has been extensively documented [113]. LPS can disrupt the intestinal and blood-brain barriers, triggering a robust inflammatory response in the brain, promoting Aβ deposition, and inducing excessive phosphorylation of tau [111, 114]. Furthermore, it upregulates the activity of APP cleaving enzyme BACE-1 and γ-secretase while reducing the activity of α-secretase, thus promoting Aβ generation. It also induces neuroinflammation in microglia cells and impairs cognitive function of AD mice in a CatB-dependent manner [115]. Meanwhile, LPS downregulates the expression of LRP-1, impairs P-glycoprotein function, and disrupts Aβ clearance through multiple pathways. LRP-1 is the primary metabolic receptor for APOE in the brain and involved in APOE-mediated lipid transport processes [116], indicating that LPS may indirectly regulate the function of APOE. Additionally, LPS induces oxidative stress and mitochondrial dysfunction through NOX2, leading to neuronal damage and synaptic loss [113].

Furthermore, an important point not to be overlooked regarding LPS is its close association with microglial activation and neuroinflammation [117, 118]. LPS serves as a receptor agonist for Toll-like receptor 4 (TLR4), which is considered one of the key receptors involved in the innate immune system of microglia [119], promoting the production of pro-inflammatory cytokines and increasing Aβ accumulation [120]. Additionally, LPS can interact with TREM2 to modulate microglial phenotype transition from anti-inflammatory to pro-inflammatory [121]. Therefore, LPS-induced neuroinflammation is also a crucial factor in promoting neurodegeneration and cognitive impairment in AD.

From the perspective of lipid metabolism, research has shown that LPS can induce the expression of microglial Cholesterol 25-hydroxylase (CH25H) [122]. CH25H is an enzyme responsible for hydroxylating cholesterol to produce oxysterol 25-hydroxycholesterol (25-HC), which is upregulated in brain tissues of AD patients, as well as in APP/PS1 and PS19 mouse brain tissues [123]. Regulation of CH25H by LPS results in increased synthesis and release of 25-HC, which in turn activates LXR gene expression and inhibits SREBP expression, enhancing cholesterol esterification and lipid droplet accumulation in astrocytes [124]. Further studies have found that LPS-mediated activation of 25-HC disrupts mouse hippocampal plasticity and memory learning function [122]. Given the evidence that Gram-negative bacteria and LPS impact various AD pathologies, Gram-negative bacteria and LPS represent attractive novel targets for AD therapy.

### Trimethylamine N-oxide

The anaerobic bacteria in the gut microbiota, such as *Clostridia* and *Enterobacteriaceae*, degrade carnitine, choline, and lecithin to produce trimethylamine (TMA), which is subsequently oxidized and reduced in the liver to generate Trimethylamine N-oxide (TMAO) [103, 125]. Elevated levels of TMAO have been found in the cerebrospinal fluid of patients with mild cognitive impairment and AD, and are associated with pathological markers of AD including tau phosphorylation, Aβ deposition, and neurodegeneration [126].

TMAO activates the NOD-like receptor protein 3 (NLRP3) inflammasome to induce inflammatory responses through SIRT3-SOD2-mtROS pathway [127]. TMAO also influences lipid and hormone homeostasis, regulates cholesterol and steroid metabolism, and reduces cholesterol reverse transport, thereby promoting the development of various diseases [128]. Furthermore, TMAO affects the tricarboxylic acid (TCA) cycle by decreasing ketone and fatty acid oxidation, thus lowering energy metabolism and inhibiting mitochondrial function. Researchers observed that TMAO promotes the downregulation of synaptic plasticity-related proteins and mTOR signaling pathway expression, leading to mitochondrial damage and superoxide production, impairing synapses, and causing a decline in mice cognitive function [129]. Decreasing plasma TMAO levels has been shown to reduce the expression of pro-inflammatory cytokines IL-2, IL-17, and TNF-α, alleviate the hippocampal inflammation in APP/PS1 mice, and improve the cognitive ability and pathological progression [130].

### Tryptophan catabolites

Tryptophan (TRP), an indispensable aromatic amino acid obtained from dietary source [131], undergoes regulation by the intestinal microbiota [132]. Through liquid chromatography metabolomics analysis, significant differences were found in tryptophan catabolites (TRYCATs) between AD patients and healthy controls, indicating a link between cognitive impairment in AD patients and dysregulated tryptophan metabolism resulting from microbial imbalance [91]. TRYCATs have been shown to stimulate neurogenesis in mice through an aryl hydrocarbon receptor (AhR)-dependent manner [133, 134]. Furthermore, AhR is intricately linked to sphingolipid metabolism, as it upregulates sphingolipid and S1P levels, thereby contributing to the maintenance of axonal myelination [135, 136]. These findings suggest a potential connection between tryptophan metabolism and sphingolipid metabolism in AD.

Indole, the principal microbial metabolite originating from tryptophan degradation in adult organisms, assumes a crucial neuroprotective role in AD by modulating inflammatory responses, preserving gut barrier integrity, and regulating immune homeostasis [137]. Notably, a diminished abundance of indole-producing bacteria, including *Firmicutes*, *Bacteroidetes*, *Actinobacteria*, and *Lactobacilli*, was observed in AD patients [91], concomitant with a significant reduction in indole levels [138]. Indole-3-propionic acid (IPA), synthesized by *Clostridium* *sporogenes*, is capable of intestinal absorption and entry into the brain, where it serves to scavenge hydroxyl radicals, reduce DNA damage, and inhibit the formation of amyloid fibrils [137]. Indole compounds exhibit neuroprotective effects due to their antioxidant, anti-inflammatory, immunomodulatory, and anti-amyloidogenic properties, gradually emerging as candidate drugs for improving neurodegenerative diseases. Relevant drug development efforts are currently underway [139]. The indole compound NC009-1 has been shown to improve cognitive deficits of 3× Tg-AD mice by upregulating the expression of APOE and tropomyosin receptor kinase A and reducing the levels of Aβ and tau in the hippocampus and cortex [140].

Besides, metabolomic analyses, both untargeted and targeted, of cerebrospinal fluid (CSF) derived from individuals with AD unveiled a specific dysregulation in the tryptophan-kynurenic acid pathway within the central nervous system. Notably, tryptophan catabolic metabolites, namely kynurenic acid and quinolinic acid, exhibited elevated concentrations in the CSF of AD patients. Moreover, these metabolites demonstrated a robust correlation with the core pathological amyloid proteins Aβ42 and phosphorylated Tau181 [141].

## Gut microbiota metabolites interact with key genes of AD lipid metabolism

**Fig. 2:**
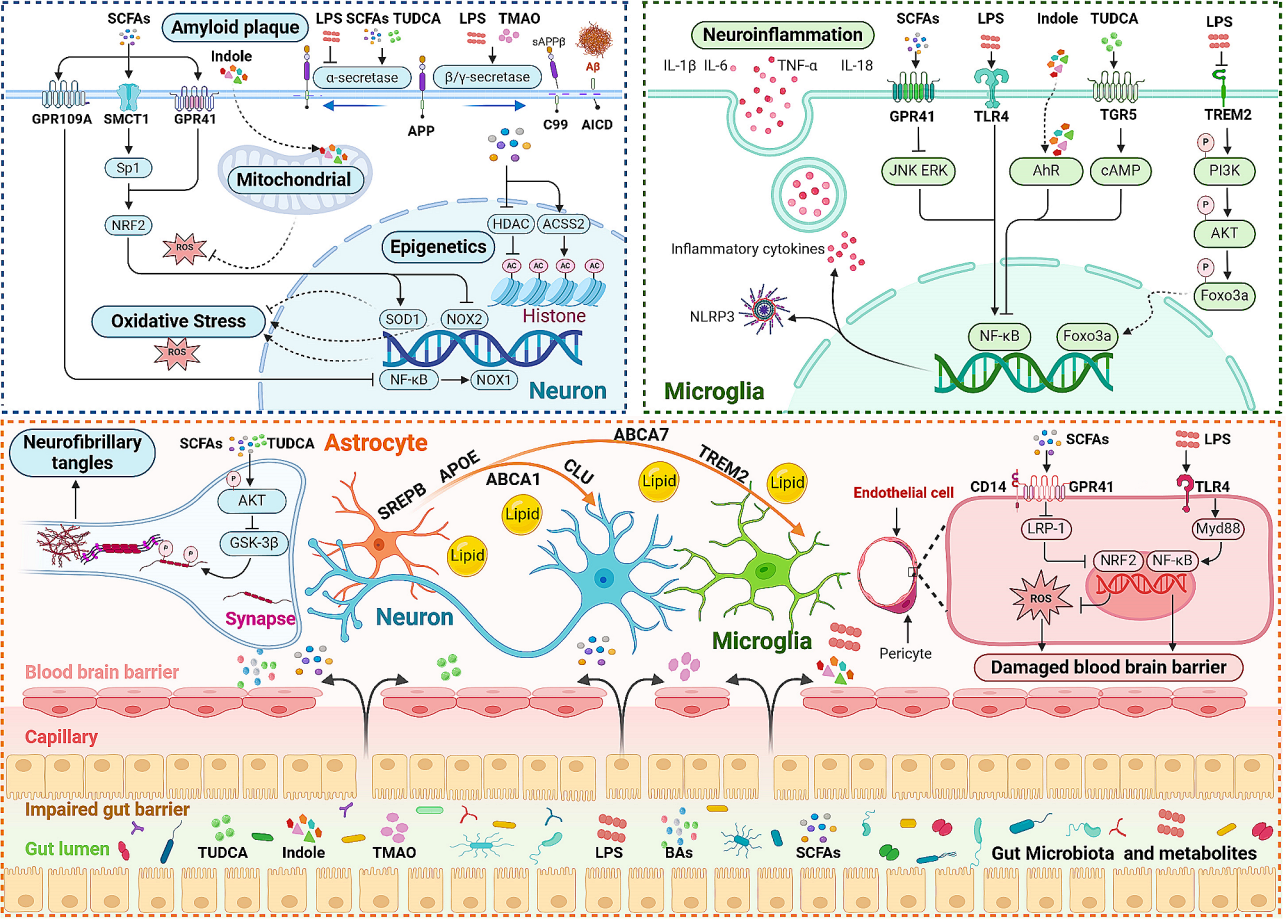
The connection mechanisms between gut microbiota and pathology of Alzheimer’s disease. The dysregulation of gut microbiota compromises the integrity of the intestinal and blood-brain barriers, allowing gut microbiota metabolites to enter the central nervous system and participate in the pathological processes of Alzheimer’s disease (AD). Astrocytes within the central nervous system synthesize lipids through key genes such as SREBP, APOE, ABCA1, CLU, ABCA7, TREM2, transferring them to neurons and microglial cells. Microbial metabolites can interact with these genes, influencing lipid homeostasis. Furthermore, gut microbiota metabolites primarily contribute to AD pathology through involvement in Aβ pathology, tau pathology, neuroinflammation, oxidative stress, mitochondrial dysfunction, and epigenetic regulation. SCFAs binding to GPR41, dependent on CD14 expression, inhibits NRF2 signaling, reducing oxidative stress in endothelial cells, maintaining the blood-brain barrier. In contrast, LPS binding to TLR4 promotes Myd88 expression, activating NF-κB transcription, releasing pro-inflammatory cytokines, damaging the blood-brain barrier. For Aβ Pathology: LPS reduces α-secretase activity, promoting APP production, while both LPS and TMAO upregulate BACE-1 and γ-secretase activities, enhancing Aβ production. SCFAs and TUDCA promote α-secretase activity and inhibit Aβ generation. Tau Pathology: SCFAs and TUDCA inhibit GSK-3β activity by promoting AKT phosphorylation, thereby suppressing tau phosphorylation, and reducing NFTs formation. Neuroinflammation: SCFAs upregulate GPR41 expression, inhibit the ERK/JNK/NF-κB pathway, reducing COX2 and IL-1β levels, alleviating neuroinflammation. TUDCA binds to the TGR5 receptor in microglial cells, increases cAMP levels, inhibits the NF-κB pathway, induces an anti-inflammatory phenotype, and mitigates inflammation. Indole reduces NLRP3 inflammasome expression through the AhR/NF-κB pathway, decreasing the release of inflammatory factors TNF-α, IL-6, IL-1β, and IL-18, inhibiting microglia-induced neuroinflammation. TREM2 activates the PI3K/AKT/Foxo3a pathway, suppressing the inflammatory response. Conversely, downregulation of TREM2 expression by LPS leads to an increased inflammatory response. LPS also activates TLR4 and NF-κB transcription, leading to the release of pro-inflammatory cytokines TNFα, IL-6, and IL-1β. Oxidative Stress and Mitochondrial Function: SCFAs binding to GPR109A blocks NF-κB signaling, reducing neuronal oxidative stress levels. SCFAs binding to sodium-coupled monocarboxylate transporter 1 (SMCT1) or activating GPR41 promotes NRF2, leading to increased SOD1 production and inhibition of NOX2, preventing excessive accumulation of neuronal ROS. Indole binds to respiratory chain complex enzymes, reducing mitochondrial electron leakage, neutralizing hydroxyl radicals, and inhibiting ROS production. Epigenetic Regulation: SCFAs inhibit HDAC activity, promoting excessive acetylation of histone H3K18, improving cognition. Alternatively, SCFAs promote histone acetylation, restoring synaptic plasticity and cognitive function in an ACSS2-dependent manner

Genome-wide association studies (GWAS) have pinpointed risk genes for AD, including apolipoprotein E (APOE), clusterin (also referred to as apolipoprotein J, CLU), and ATP-binding cassette sub-family A, members 1 and 7 (ABCA1/7). These genes play a central role in lipid metabolism and their alterations contribute to the development of lipid metabolism disorders in AD [142–144]. Furthermore, SREBP-2 is also a key regulator of cholesterol metabolism and is genetically associated with an altered risk of AD [145]. Numerous studies indicates that the gut microbiota and its metabolites interact with these key lipids genes and participate in the pathological progression of AD (Fig. 2). Therefore, this section focuses on summarizing the above-mentioned content.

### APOE

APOE functions as a lipid transport protein with a crucial role in the central nervous system, and its significance and functions in the context of Alzheimer’s disease have been extensively documented in numerous research studies [146–149]. APOE facilitates the transport of cholesterol and lipids between astrocytes and neurons by interacting with the low-density lipoprotein receptor (LDLR) and LDLR-related protein 1 (LRP1). This mechanism is pivotal in synaptic formation and tissue repair processes [147]. With its three main isoforms, APOE exhibits a range of effects. APOE2 exhibiting a protective effect, APOE3 playing a neutral role, while the APOE4 allele increases the risk of developing AD by approximately 12 times in homozygous individuals [150]. Consequently, APOE4 is considered the most potent risk factor for late-onset Alzheimer’s disease (LOAD) [114, 151, 152].

In-depth analyses of the gut microbiota in AD mice with different APOE genotypes, utilizing 16S rRNA sequencing and fecal metabolomics, have revealed correlations between APOE genotypes and the abundance of gut microbiota. Specifically, APOE2 genotype mice displayed higher levels of *Ruminococcaceae* and *Prevotellaceae*, bacterial families involved in SCFAs production. This is thought to contribute to the protective effect of the APOE2 genotype against AD. In APOE4 genotype AD mice, an increase in *Lachnospiraceae* and *Deferribacteraceae*, and a decrease in *Bacteroidaceae* were observed, accompanied by reduced concentrations of SCFAs and their precursors [153]. These findings suggest that APOE genotypes influence the composition of the gut microbiota and the generation of metabolites in AD mice. Changes of microbiota and metabolites induced by different APOE genotypes may play an important role in the impact of APOE genes on AD. Carriers of the APOEε4 allele often experience disturbances in CNS cholesterol homeostasis, and APOE4 mice also exhibit abnormal cholesterol levels and lipid metabolism disruptions [153]. This suggests that the gut microbiota and SCFAs may influence CNS cholesterol levels by affecting APOE gene.

Moreover, the microbiota and its metabolites also influence APOE genes. For instance, the microbiota-produced secondary bile acid TUDCA reduce the expression of APOE in the hippocampus and frontal cortex, inhibiting the production and accumulation of Aβ [154]. In the presence of melatonin, APOE4’s characteristics shift from promoting amyloid fibril formation to inhibiting it [137]. Additionally, the gut microbiota reduces neuroinflammation, tau pathology, and neurodegeneration in an APOE genotype-specific manner [100]. These findings highlight the significant interplay between the microbiota, its metabolites, and APOE genes in AD.

### TREM2

TREM2 is a transmembrane receptor of the immunoglobulin superfamily specifically expressed in microglial cells of the central nervous system. It plays a role in microglial proliferation, transportation, phagocytic functions, and inflammatory responses [155, 156]. When functional loss mutations, such as R47N, R62H, and D87N, occur in TREM2, the risk of developing LOAD increases. These mutations result in reduced cholesterol clearance by microglial cells, decreased uptake of CLU, LDL, and Aβ, exacerbating cholesterol lipid accumulation, amyloid pathology, and neuronal damage [81, 157–159].

Research has reported a close association between bacterial anionic LPS and TREM2. LPS bind to TREM2 [160], promoting the transition of microglial cells from an anti-inflammatory phenotype to a pro-inflammatory phenotype [161]. A study in BV2 cells confirmed that LPS reduces TREM2 expression, promotes the translocation of Foxo3a from the cytoplasm to the nucleus, triggering an inflammatory response. Conversely, upregulation of TREM2 expression can activate the PI3K/AKT/Foxo3a axis, inhibiting pro-inflammatory factors and shifting microglial cells toward an M1 phenotype [162]. Moreover, TREM2 facilitates the expression of Sirtuin3, suppressing LPS-induced oxidative stress and neuroinflammation [163]. The deletion of TREM2 results in a substantial increase in inflammatory mediators, including Cxcl10, Rac2, and Casp1, upon exposure to LPS, thereby fostering the initiation of inflammatory responses [164].

### ABCA1/7

ATP-binding cassette subfamily A (ABCA) belongs to the ATP-binding cassette transporter family, mediating the export of cholesterol and phospholipids in the brain. Notably, rare variants of ABCA1 and ABCA7 have been identified as risk genes for AD [64]. ABCA1 transports cholesterol, phospholipids, and other lipid molecules to lipoprotein carriers [165] and facilitates the distribution of cholesterol from astrocytes to neurons, protecting cells from the toxic effects of excessive free cholesterol [166]. Simultaneously, ABCA1 is responsible for lapidating the APOE protein in the brain. The deficiency of ABCA1 results in impaired APOE lipidation, leading to increased Aβ plaque load, while overexpression of ABCA1 can reduce Aβ deposition [167, 168]. Research indicates that the gut microbiota metabolite butyrate activates the expression of ABCA1, promoting cholesterol efflux, thereby improving lipid metabolism in APOE^−/−^ mice [169]. TMAO has been demonstrated to downregulate the expression of ABCA1, promoting cholesterol accumulation [170, 171].

ABCA7, sharing 54% homology with ABCA1, is currently recognized for its involvement in facilitating lipid transport from neurons to glial cells, providing protection to neurons against oxidative lipid damage. Ongoing research suggests that ABCA7 also plays a crucial role in cholesterol transport and phagocytosis by microglial cells [172, 173]. Conversely, the absence or functional impairment of ABCA7 may intensify fatty acid consumption, disturb brain inflammatory responses, reduce microglial phagocytic function, and elevate Aβ levels [49, 174]. Furthermore, in the setting of LPS-induced brain inflammation, ABCA7 haplodeficiency enhances the production of EPA during the acute inflammatory phase, thereby facilitating the resolution of acute inflammation [175].

### CLU

CLU, alternatively referred to as apolipoprotein J, demonstrates predominant expression within the central nervous system and holds a pivotal role in cholesterol and lipid transport. Genome-wide association studies have established a significant association between CLU allelic variations and the susceptibility to AD. In comparison to control counterparts, AD patients manifest notably increased CLU levels, and empirical evidence supports the direct interaction of CLU with Aβ, thereby facilitating the formation of Aβ fibrils [176].

Researchers have observed that the deletion of the CLU gene alters the abundance and composition of the gut microbiota, characterized by an increase in *Bacteroidetes* and a decrease in *Firmicutes*, indicating a close association between CLU and the gut microbiota [177]. Increased TMAO levels induce elevated CLU protein expression, consequently triggering neuroinflammation and the production of β-secretase and βCTF in the hippocampus, resulting in Aβ deposition. Conversely, the inhibitor 3,3-Dimethyl-1-butanol reduces TMAO levels, inhibits CLU protein expression, and improves hippocampal inflammation and cognition [130]. Treatment with *Lactobacillus plantarum* has been demonstrated to inhibit the production of gut microbiota-derived TMA and synthesis of TMAO, leading to reduced CLU expression, alleviation of cognitive impairment, and attenuation of neuropathology [178]. Currently, limited evidence supports the connection between CLU and the gut microbiota, necessitating further exploration of their relationship and the roles they play in the progression of AD. Considering the reduction of TMAO production via microbial modulation may offer a potential avenue for mitigating CLU-mediated lipid homeostasis in AD.

### SREBP-1/2

Sterol regulatory element-binding proteins (SREBPs) function as transcription factors, modulating the expression of genes associated with lipid synthesis. There are three isoforms of SREBP in mammals: SREBP1a, contributing to overall lipid synthesis and growth; SREBP1c, associated with fatty acid synthesis and energy storage; and SREBP2, playing a role in cholesterol regulation [179, 180]. The overexpression of SREBP-2 in APP/PS1 mice has been reported to exacerbate amyloid pathology and neuronal death, accompanied by a decline in cognitive abilities, while the inhibition of SREBP-2 alleviates amyloid burden in AD mice [181, 182]. Additionally, another study validates that SCFAs can enhance the gene expression of SREBP2, consequently facilitating hepatic absorption of serum cholesterol and augmenting the excretion of fecal bile acids [183]. In 3×Tg-AD mice, elevated expression of SREBP1c results in increased cholesterol synthesis. Treatment with the probiotic SLAB51 reduces the protein expression of SREBP1c in the brain and liver, thereby improving the lipid profile and cognitive function [46].

## Molecular mechanisms of gut microbiota regulation of AD lipids and pathology

The mechanistic links between lipid metabolism dysregulation and AD include amyloid pathology, tau pathology, neuroinflammation, oxidative stress, and mitochondrial dysfunction. Additionally, the gut microbiota can directly or indirectly participate in the pathological mechanisms of AD by influencing lipid metabolism (Fig. 2). This section focuses on presenting evidence for the role of gut microbiota in the mechanisms of AD lipids and pathology.

### Amyloid pathology

The abnormal deposition of amyloid is a central pathological feature of AD [184]. Aβ, a peptide with a length of 36–43 amino acid residues, is generated through consecutive cleavages of APP by β-secretase (mainly BACE1) and γ-secretase [185, 186], with Aβ40 and Aβ42 being the most common in AD. The activities of β-secretase and γ-secretase are highly dependent on the lipid levels in the membrane, emphasizing the close connection between Aβ pathology and lipids [186]. Certainly, the generation of Aβ takes place within specialized membrane microdomains called lipid rafts, which are enriched with cholesterol, phospholipids, and sphingolipids [186, 187]. Increased brain cholesterol levels boost the activity of β-secretases and γ-secretases within lipid rafts, suppress α-secretase activity, and stimulate the generation of harmful Aβ [185]. Aggregates of Aβ can directly engage with cell membrane lipids and cholesterol, disrupting membrane integrity and permeability, fostering Ca^2+^ influx, ultimately resulting in neuronal death [188].

Research indicates that BAs produced by gut microbiota increase the permeability of both the intestinal and blood-brain barriers [189], facilitating the entry of peripheral cholesterol into the central nervous system. The elevated brain cholesterol directly binds with APP, promoting the insertion of APP into the lipid raft phospholipid layer and consequently increasing the production of Aβ. Moreover, BAs downregulate the expression of cholesterol-metabolizing enzyme CYP46A through the farnesoid X receptor (FXR), promoting cholesterol accumulation and increasing Aβ production [190]. LPS, similar to BAs, have been shown to enhance the expression of APP, reduce the activity of α-secretase, upregulate the activity of APP-cleaving enzyme BACE-1 and γ-secretase, promoting Aβ generation [115]. Furthermore, the gut microbiota metabolite TMAO has been implicated in promoting Aβ accumulation in AD mice by increasing the activity of β-secretase through elevating CLU levels, leading to cognitive impairment [130].

### Tau pathology

Tau pathology is initiated by the aggregation of phosphorylated tau into oligomers, protofibrils, and filamentous NFTs, leading to synaptic damage and neurodegeneration [191, 192]. Hence, tau pathology is also considered a major driving factor in the neurodegenerative changes associated with AD [1]. Evidence suggest that cholesterol and lipoprotein particles impact the excessive phosphorylation of tau in neurons [193]. Further investigations reveal that CE induce tau phosphorylation through the CE-proteasome-tau axis. Inhibiting cholesterol synthesis in astrocytes significantly reduces the neuronal tau burden [67, 193]. These studies confirm that disrupted cholesterol metabolism influences tau pathology of AD.

In a recent study, researchers subjected tau transgenic mice expressing different APOE isoforms to GF housing and antibiotic interventions. They found that modulation of gut microbiota reduced tau pathology, and neurodegeneration in an APOE genotype-dependent manner. Supplementation with SCFAs exacerbated tau pathology in GF-TE4 mice [100]. Additionally, increased abundance of the *Bacteroides fragilis* upregulates the PUFA metabolites PGE1 and 12-HHTrE, triggering C/EBPβ/AEP signaling activation, and exacerbating tau pathology [194]. *Lactobacillus plantarum* DP189 increases the presence of *Firmicutes* and, through the modulation of the PI3K/AKT/GSK3β pathway, decreases the levels of GSK3β, effectively suppressing tau hyperphosphorylation [195]. TUDCA inhibits the activation of AKT and suppresses GSK3β activity, reducing tau hyperphosphorylation and microglial activation, ultimately improving AD pathology [107].

### Neuroinflammation

Neuroinflammation is recognized as a crucial characteristic of brain tissue in AD patients, orchestrated predominantly by microglial cells and astrocytes [196]. In this context, misfolded and aggregated proteins bind to pattern recognition receptors, such as Toll-like receptors (TLRs), located on the surfaces of microglial and astrocytic cells, initiating inflammatory responses. Acute inflammatory responses are believed to contribute to the clearance of Aβ, restoring tissue homeostasis. However, sustained inflammation induced by persistent Aβ stimulation and immune activation leads to the continuous release of pro-inflammatory factors and inflammatory mediators, increasing Aβ deposition and promoting the formation of neurofibrillary tangles, thereby exacerbating neuronal and synaptic damage and contributing to the progression of AD [197]. Indeed, the critical role of neuroinflammation in AD has been reported as early as the year 2000 [198].

Current research has elucidated the impact of the gut microbiota on neuroinflammation in AD. The potential connection between the microbiota and AD may involve an imbalance in gut homeostasis, characterized by an increase in inflammatory responses and a reduction in anti-inflammatory microbes [196, 199]. Microbiota analysis in AD patients reveals an increased abundance of pro-inflammatory bacterial groups and a decrease in butyrate-producing taxa such as *Butyrivibrio*, *Eubacterium*, *Clostridium sp*. *strain SY8519*, and *Faecalibacterium prausnitzii* [200]. In 3×Tg mice, similar phenomenon is observed, where beneficial anti-inflammatory bacterial groups, such as *Firmicutes* and *Cyanobacteria*, gradually decrease with age, while pro-inflammatory taxa, including *Bacteroidetes* and *Ruminococcus*, significantly increase [45]. Furthermore, there is a close association between gut microbiota and their metabolites with the activation of glial cells [201, 202]. Gut microbiota can stimulate glial cells to participate in the neuroinflammatory response in AD [203, 204]. For instance, antibiotic treatment in APP/PS1 mice has been shown to reduce neuroglial reactivity, thereby alleviating Aβ plaque deposition [37, 205]. Acetate has been shown to upregulate the expression of GPR41 in microglial cells, inhibit the ERK/JNK/NF-κB pathway, reduce the levels of COX2 and IL-1β, counteract neuroinflammation in APP/PS1 mice, and improve cognitive abilities [94]. LPS, by activating the microglial cell surface TLR4 receptor, triggers downstream NF-κB transcription, leading to the activation of numerous pro-inflammatory factors such as TNFα, IL-6, and pro-IL-1β [114]. Indole acts through the AhR-NF-κB pathway, decreasing the expression of NLRP3 inflammasomes, reducing the release of inflammatory cytokines TNF-α, IL-6, IL-1β, and IL-18, alleviating inflammation, and improving cognitive and behavioral abilities in APP/PS1 mice [138].

The gut microbiota may influence neuroinflammatory responses in AD by affecting lipid metabolism. In brief, activation of the C/EBPβ/AEP signaling pathway by the gut microbiota in the brains of 3×Tg mice upregulates mRNA transcription for inflammatory enzymes related to AA [206], resulting in increased release of AA metabolites such as prostaglandin E2 (PGE2), thromboxane B2, LKB4, and 12-HHT. This activation triggers microglial cell activation and intensifies neuroinflammation, ultimately worsening cognitive impairment. Further investigations revealed that specific gut bacteria, including *Bacteroides intestinalis*, *Bacteroides fragilis*, and *Bacteroides xylanisolvens*, produce AA metabolites and contribute to AD neuroinflammatory responses. Conversely, a decrease in the abundance of anti-inflammatory SCFAs, due to reduced levels of *Butyrivibrio* and *Eubacterium*, exacerbates chronic neuroinflammation, elevates APP and tau expression levels, and contributes to AD pathogenesis [45]. In a recent study, the authors identified that *Bacteroides fragilis* and its metabolites, 12-hydroxy-17-carbon-triene acid and PGE2, mediate PUFA metabolism in the gut microbiota of AD. This activation leads to microglial cell induction of AD-like pathology and cognitive impairments [194], providing compelling evidence for the role of gut microbiota in regulating AD lipid metabolism and neuroinflammation.

### Oxidative stress

Oxidative stress refers to the imbalance in cellular redox equilibrium caused by the accumulation of reactive oxygen species (ROS) and reactive nitrogen species (RNS). ROS play a vital role in normal physiological conditions by participating in signaling pathways and transcriptional activation. Nevertheless, continuous accumulation of ROS, coupled with compromised cellular antioxidant capacity, can result in the assault on cellular macromolecules such as lipids, proteins, and DNA. This process leads to lipid peroxidation, protein oxidation, and nucleic acid damage [207, 208]. Concurrently, inflammation and mitochondrial dysfunction disturb redox balance, fostering the generation and release of ROS [209]. The elevation of ROS and oxidative stress is recognized as one of the hallmarks of AD.

The central event of oxidative stress is lipid peroxidation, and the brain, rich in polyunsaturated fatty acids, is susceptible to oxidative stress due to lipid peroxidation [210]. SCFAs play a crucial role in regulating lipid peroxidation. For example, butyrate enhances fatty acid oxidation, electron transport chain, and oxidative stress gene expression, while propionate interacts with fatty acid receptors, upregulating lipoprotein lipase to promote lipid synthesis [211, 212]. Acetate is involved in regulating cholesterol metabolism and adipogenesis [213]. Thus, existing evidence supports the role of gut microbiota in mediating oxidative stress alterations in AD [208]. Propionate bind to the free fatty acid receptor GPR41 in brain endothelial cells, inhibiting the expression of LRP-1 through a CD14-dependent mechanism, and protecting the blood-brain barrier from oxidative stress through NRF2 signaling [95]. Butyrate act as ligands for GPR109A, and by activating GPR109A, they block the NF-κB signaling pathway [212], a crucial pathway in oxidative stress in AD [214, 215]. Additionally, cells uptake butyrate through the sodium-coupled monocarboxylate transporter 1 (SMCT1), leading to the generation of Sp1. This activation of Sp1 stimulates NRF2, thereby promoting the production of SOD1 and suppressing NOX2, preventing the excessive accumulation of reactive ROS in neurons [216]. Indole derivative IPA, an effective scavenger of hydroxyl radicals, protects central neurons from oxidative damage by reducing DNA damage and lipid peroxidation [212].

### Mitochondrial dysfunction

Various pieces of evidence indicate that mitochondrial dysfunction is an early event in the pathogenesis of AD, including alterations in mitochondrial structure, respiratory dysfunction, reduced ATP generation, impaired dynamics, and elevated mitochondrial-associated oxidative stress [192, 217, 218]. Mitochondrial dysfunction leads to the release of cytochrome c, which activates Caspase-9-dependent neuronal apoptosis, disrupting Ca^2+^ homeostasis and triggering neuronal death. The association between the gut microbiota and mitochondria has been extensively documented over an extended period [219, 220]. Microbial metabolite N6-carboxymethyllysine (CML) mediates ROS burst, damaging mitochondrial activity and ATP storage in microglial cells [221]. Butyrate enhances mitochondrial biogenesis in astrocytes by upregulating the expression of PGC-1A, contributing to improved mitochondrial function and enhanced cognitive abilities in AD mice [222]. IPA and indole-3-propionamide (IPAM) exert neuroprotective effects by mitigating mitochondrial electron leakage and neutralizing hydroxyl radicals. Moreover, indole compounds, including IPA and IPAM, permeate the mitochondrial membrane, binding to the rate-limiting phosphorylation site on respiratory chain complex I. This action serves as an energy metabolism stabilizer, leading to a reduction in ROS production and contributing to neuroprotection [137].

### Epigenetic regulation

Epigenetic regulation encompasses processes such as histone modification, DNA methylation, chromatin remodeling, and non-coding RNA regulation, all of which have been demonstrated to play a crucial role in neurodegenerative diseases [92, 93]. It is well-established that the expression levels of key proteins implicated in AD, including APP, BACE1, PS1, and APOE, are subject to epigenetic regulation. Therefore, the imbalance in epigenetic regulation may underlie the aberrant expression of genes associated with synaptic plasticity and memory in AD [223]. Histone modification is a common form of epigenetic regulation in AD, influencing the stability of nucleosomes, chromatin-mediated processes to participate in the regulation of gene expression. Histone modification encompasses acetylation, methylation, ubiquitination, among others, with acetylation playing a crucial role in AD [224]. Research has revealed a reduction in H4K12 histone acetylation levels in aged mice, leading to defects in the expression of learning and memory-related genes [225]. Histone acetylation is regulated by histone acetyltransferases (HATs) and histone deacetylases (HDACs). In AD mice and patients, the expression of HDAC2 increases with age [226]. HDAC2 overexpression reduces dendritic spine density, impairs neural plasticity and memory function, and suppressing or downregulating HDAC expression effectively restores cognitive function in AD animals [227].

Butyrate is described as a HDAC inhibitor, improving memory function in APP/PS1 mice by enhancing hippocampal histone acetylation [96]. Acetate enhances histone H3K18 over-acetylation and epigenetic regulation of BDNFPII and PIV promoter regions, leading to increased expression of BDNF and improvement in cognition [97]. Furthermore, acetate supplementation, as demonstrated in 5×FAD mice, enhances histone acetylation, restoring synaptic plasticity and cognitive function in an ACSS2-dependent manner [228]. Studies have reported that the immature phenotype of microglia in GF mice arises from epigenetic markings of key mitochondrial genes by H3K4me3 and H3K9ac, accompanied by intracellular fatty acid and lipid depletion. Acetate, driving microglial maturation and metabolic homeostasis, can rescue the impaired microglial function in GF mice. In the pathological context of AD, acetate exhibits inhibitory effects on microglial phagocytic function, leading to increased Aβ burden in 5×FAD mice [99].

### Targeting gut microbiota and lipids for the treatment of AD

**Fig. 3:**
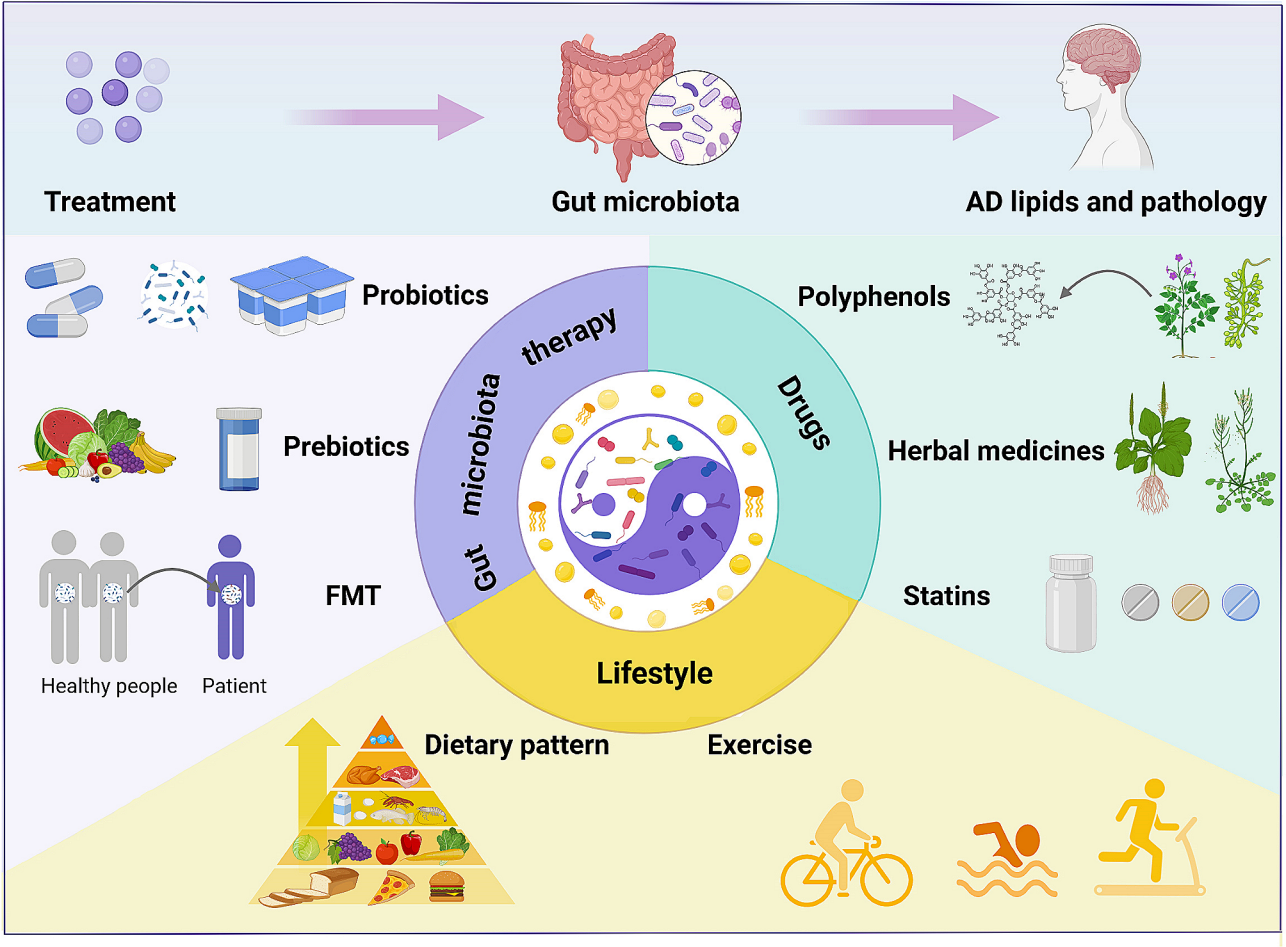
Potential interventions in the gut microbiota to regulate lipid balance and mitigate pathological progression in Alzheimer’s disease. Current evidence suggests that interventions such as gut microbiota-based therapies (probiotics, prebiotics, fecal microbiota transplantation), pharmacological treatments (polyphenols, herbal remedies, statins), and lifestyle modifications (dietary patterns, exercise) can target the gut microbiome. These interventions promote gut microbiota and lipid homeostasis, ultimately enhancing cognitive function. These measures hold promise as potential strategies for preventing and treating the progression of Alzheimer’s disease

Given the evidence supporting the role of gut microbiota and lipid metabolism in the pathological progression of AD, it becomes crucial to understand how modulating the levels of microbiota and metabolites to improve AD pathology. Therefore, this section summarizes the primary preventive and therapeutic interventions aimed at regulating gut microbiota and lipid metabolism (Fig. 3).

### Gut microbiota-based therapy

#### Probiotics

Probiotics, as live microbial supplements, have been shown to effectively improve gut microbiota balance and provide therapeutic benefits for patients with AD [229]. Studies have demonstrated the therapeutic effects of SLAB51 on AD, it improves glucose homeostasis, reduces tau phosphorylation [230], increases SCFAs such as acetate, propionate, and butyrate, while decreasing inflammation and Aβ deposition [231]. It activates a SIRT1-dependent mechanism, reducing oxidative stress in the brains of AD mice [232]. Another study found that SLAB51 inhibits cholesterol biosynthesis, lowers the ω-6/ ω-3 fatty acid ratio, improves neuroinflammation and oxidative stress, ultimately reducing Aβ and tau aggregation, and slowing down AD progression [46]. Candida rugosa lipase (CRL) has been reported to increase the abundance of *Acetatifactor* and *Clostridiales vadin BB60* in the gut, enhancing lipid hydrolysis and maintaining unsaturated fatty acid homeostasis, leading to reduced neuroinflammation and cognitive deficits in APP/PS1 mic [233]. *Lactobacillus plantarum* DP189 regulates gut microbiota dysbiosis and inhibits tau hyperphosphorylation, as reported [195]. Additionally, it suppresses TMA production and TMAO synthesis, thereby reducing CLU expression and alleviating neuroinflammation and neuropathological defects in APP/PS1 mice [233]. The probiotic VSL#3 efficiently reduces serum prostaglandin and deoxycholic acid levels, ameliorating intestinal inflammation and permeability. Nonetheless, its influence on brain plaque deposition, cytokine levels, and gliosis appears to be relatively limited [234]. Synthesizing this evidence with findings from other studies implies that combining probiotics with exercise could represent a more promising therapeutic strategy for enhancing cognitive function [235, 236]. In summary, probiotics modulate gut microbiota composition and lipid metabolism homeostasis, exerting positive effects on brain inflammation, oxidative stress, Aβ pathology, and tau pathology. Therefore, by regulating the microbial composition through probiotics, new preventive and therapeutic options for AD are proposed.

#### Prebiotics

Prebiotics, composed of nondigestible oligosaccharides, human milk oligosaccharides, and soluble, fermentable fibers, serve as an alternative to probiotic supplements. They effectively enhance beneficial bacteria such as *Bifidobacteria* and *Lactobacilli*, improving cognitive impairment in APP/PS1 mice through the gut-brain axis [237, 238]. The neuroprotective effects of prebiotics make them a potential oral formulation for the prevention and treatment of AD [239]. Fructooligosaccharides (FOS) demonstrate beneficial effects in APP/PS1 mice [190], and prebiotic mannan oligosaccharide (MOS) reshapes the gut microbiota, maintains intestinal barrier integrity, increases SCFAs production, inhibits neuroinflammation and oxidative stress, effectively alleviating cognitive and behavioral deficits in 5×FAD mice [240]. In conclusion, supplementing prebiotics to modulate microbial composition and function holds promise for improving AD; however, further research is needed to explore the applicability of prebiotics in AD treatment.

#### Fecal microbiota transplantation

Fecal microbiota transplantation (FMT) involves transferring a sample of healthy donor fecal microbiota into the gut of a patient or diseased animal to restore gut microbial health and improve disease treatment. Studies have shown that transplanting fecal microbiota from wild-type mice to ADLP^APT^ mice effectively ameliorated Aβ plaques, neurofibrillary tangles, glial reactivity, and cognitive impairment, suggesting that restoring gut microbial homeostasis through FMT may have beneficial effects on AD treatment [40]. Another study confirmed that fecal transplantation from WT mice increased the abundance of *Bacteroidetes*, reduced *Proteobacteria* and *Verrucomicrobia* in the gut of APP/PS1 mice, increased butyrate levels, and significantly improved pathological features such as Aβ accumulation, synaptic dysfunction, neuroinflammation, and cognitive deficits [241]. Intervention with FMT from WT mice modulate glycerophospholipid metabolism in APP/PS1 mice, leading to an amelioration of Aβ pathology and neuroinflammation [43]. Conversely, transplanting gut microbiota from AD mice impaired memory function and neurogenesis in wild-type mice [242, 243]. Although the specific functions of the gut microbiota in these contexts are not yet fully elucidated, based on these results, healthy gut microbiota transplantation appears to exhibit a positive role in AD pathology. Table 1 summarizes current research on the gut microbiota-based therapy in improving AD lipid metabolism and pathological features.

**Table 1:** Current research on the gut microbiota-based therapy in improving AD lipid metabolism and pathological features

Intervention	Model	Alterations of GM	Alterations of lipids	Main effects	Reference
VSL#3	APP NL-GF mice	*Verrucomicrobia*↑, *Actinobacteria*↑, Taurodeoxycholic acid↓	PGE2↓, 6-keto PGF1α↓, PGF2α↓, AA↑	Regulate inflammation response	[234]
*L. plantarum*	APP/PS1 mice	TMA↓, TMAO↓	CLU↓	Alleviate neuroinflammation, reduce hippocampal Aβ levels, improve cognitive	[178]
SLAB51	3×Tg-AD mice	*Bifidobacterium spp.*↑, *Campylobacterales*↓, Acetic acid↑, Propionic acid↑, Butyric acid↑	High-density lipoprotein cholesterol↑, stearic acid↑, heptadecanoic acid↑, low-density lipoprotein cholesterol↓, oxysterol 27-hydroxycholesterol↓, total cholesterol↓, ω-6/ ω-3 PUFAs ratio↓	Improve glucose metabolism, ameliorate lipid metabolism disorders, alleviate inflammatory responses, reduce cerebral oxidative stress, decrease Aβ deposition, and enhance cognition	[46, 230–232]
*Bifidobacterium breve* strain A1	Aβ injection mice	*phylum Actinobacteria*↑, *family Bifidobacteriaceae*↑, *family doribacteracea*e↓, *Lachnospiraceae*↓, acetate↑	SCFAs↑	Suppress inflammation and immune response, enhance cognitive,	[244]
NK46	5×FAD-Tg mice	*Prevotellaceae*↑, *Ruminococacea*↓, *Lachnospiraceae*↓, *Helicobacteriaceae*↓, *Pseudomonadaceae*↓, LPS↓	SCFAs↑	Inhibit inflammatory responses, reduce Aβ production and accumulation, alleviate memory and cognitive decline	[245]
Human *Lactobacillaceae*	APP/PS1 mice	*Bacteroidetes*↑, *Staphylococcus*↑, *Acinetobacter↑, Weissella*↑, *Butyricicoccus*↑, *Sphingobacterium*↑, *Proteobacteria*↓, *Desulfobacterota*↓, *Patescibacteria*↓, *Eisenbergiella*↓	Malondialdehyde↓	Reduce oxidative stress, attenuate microglia activation, improved the cognitive deficiencies	[246]
*Bifidobacterium Lactis* Probio-M8	APP/PS1 mice	*Desulfovibrionaceae*↑, *Oscillospira*↑, *Coprococcus*↑, *Clostridiales*↑, *acidifaciens*↑, *Adlercreutzia*↓, *Lactobacillus*↓,*Streptococcus*↓	fatty acid biosynthesis↑, SCFAs↑	Reduced Aβ plaque burden, protected against gut microbiota dysbiosis, alleviate cognitive impairment	[247]
*Clostridium butyricum*	APP/PS1 mice	*Alloprevotella*↑, *Deferribacteres*↓, *Helicobacteraceae*↓, *Helicobacter*↓, butyrate↑	SCFAs↑	Ameliorated microglia activation, neurodegeneration, Aβ deposition and cognitive deficits	[248]
Mannan oligosaccharide	5×FAD-Tg mice	*Prevotella*↑, *Oscillospira*↑, *Lactobacillus*↑, *Helicobacter*↓, butyrate↑,LPS↓	SCFAs↑	Ameliorated microglia activation, neurodegeneration, Aβ deposition and cognitive deficits	[240]
Morinda officinalis	APP/PS1 mice	*Arthrobacter*↑, *Phycicoccus*↑, *Streptococcus*↑, *Akkermansia*↑, *Blautia*↑, *Ruminococcus*↑, *Coprococcus*↑, *Allobaculum*↑, *Dehalobacterium*↑, *Methanolinea*↑, *Candidatus Methanoregula Lactobacillus*↑, *Allobaculum*↑, *Lactobacillaceae*↑, *Lachnospiracea*e↑, *Mucispirillum*↓, *Odoribacte*r↓, *Rikenella*↓, *Faecalibacterium*↓, *Alistipes*↓, *Parabacteroides*↓, *Anaerotruncus*↓	linolelaidic acid↑, LysoPC↑, LysoPE↑, 15(S)-hydroxyeicosatrienoic acid↓	Reduce neuronal apoptosis, improve memory	[249]
FMT	APP/PS1 mice	*Bacteroidetes*↑, *Proteobacteria*↓, *Verrucomicrobia*↓, butyrate↑	SCFAs↑	Relieved cognitive deficits, Aβ accumulation, synaptic dysfunction and neuroinflammation	[241]
FMT	APP/PS1 mice	*/*	1-stearoyl-2-hydroxy-sn-glycero-3-phosphocholine↓, PC (16:0/16:0) ↓	Decreased Aβ plaque, proliferation and activation of astrocyte and microglia	[43]

**Abbreviations**: AA = Arachidonic acid; AD = Alzheimer’s disease; Aβ = amyloid β; CLU = Clusterin; FMT = Fecal microbiota transplantation; LPS = Lipopolysaccharides; PC = Phosphatidyl choline; PGE2 = Prostaglandin E2; PGF1α = Prostaglandin f1alpha; PGF2α = Prostaglandin F2alpha; SCFAs = Short-chain fatty acids; TMA = Trimethylamine; TMAO = Trimethylamine N-oxide

### Pharmaceutical formulation

### Polyphenols

Polyphenols, natural compounds found in fruits and vegetables, possess antioxidant and anti-inflammatory properties. It has been demonstrated that they improve the pathological processes of AD by modulating microbiota homeostasis, mitochondrial function, oxidative stress, and inflammatory responses [250]. Oral administration of 200 mg/kg hawthorn flavonoid (HF) increased the proportion of *Dubosiella* and *Alloprevotella*, reversing gut microbiota and metabolic disturbances in AD mice. This led to elevated levels of docosapentaenoic acid (DPA), sphingolipids, and PC, significantly ameliorating cognitive deficits, Aβ accumulation, and abnormal activation of hippocampal astrocytes in AD mice [251]. Curcumin reduced the abundance of *Prevotellaceae*, *Bacteroides*, and *Escherichia/Shigella* in the gut of APP/PS1 mice. In BV2 microglial cells, it upregulated the expression of TREM2, alleviating neuroinflammation and amyloid plaque burden, thereby enhancing cognition [252, 253]. Bilberry anthocyanins (BA) lowered serum and brain LPS levels, increased SCFAs in feces, induced microglial phagocytosis of Aβ through the CD33/TREM2/TYROBP signaling pathway, alleviated hippocampal neuroinflammation, and reversed cognitive impairments in APP/PS1 mice [254]. In conclusion, the interaction of polyphenols with the gut-brain axis enables them to influence the central nervous system and exert neuroprotective activity. Further development of their therapeutic potential in AD is warranted.

#### Herbal medicines

Herbal medicines (HMs), also known as botanical medicines or phytomedicines, refer to plant-derived materials or preparations with therapeutic or other human health benefits. Studies have reported that the chemical substances in herbal medicines can be transformed by gut microbiota into metabolites, thereby improving the composition, functional impairments, and associated pathological progress of the gut microbiota. The regulatory effects of herbal medicines on the gut microbiota have also been applied in AD [255]. Patchouli alcohol (PA) has been demonstrated to effectively inhibit pro-inflammatory microbial groups such as *Bacteroides*, *Klebsiella*, *Bilophila, Proteobacteria*, and *Enterobacteriaceae*. It enhances the abundance of anti-inflammatory microbial groups, such as *Firmicutes* and *Lactobacillus*, suppresses the activation of the C/EBPβ/AEP pathway, alleviates Aβ plaque deposition, tau hyperphosphorylation, and neuroinflammation, ultimately improving cognitive deficits in TgCRND8 mice [256]. Schisandra chinensis (S. chinensis) improves learning and memory abilities in AD rats by increasing SCFAs levels and alleviating neuroinflammation [257]. Alpinae Oxyphyllae Fructus (AOF) has been proven to regulate TREM2 and mitigate LPS-induced neuroinflammation, promoting a beneficial M2 phenotype in microglial cells and ameliorating cognitive impairments in mice [258, 259]. Epimedii Folium and Curculiginis Rhizoma, extracts of Horny Goat Weed and Xianmao, enhance TREM2 protein expression in the hippocampus by reducing TNF-α and IL-1β, regulating the transformation and activation of microglial cells, thus improving LPS-induced cognitive impairments [260]. In addition, Pyrolae herba (PH) regulates TREM2 expression, inhibits LPS-induced neuroinflammation, and alleviates cognitive impairments [261]. The therapeutic effects of herbal medicine on AD are highly complex, involving multiple aspects, with gut microbiota and lipid metabolism being just one facet. The precise therapeutic mechanisms remain to be elucidated.

#### Statins

Statins inhibit HMG CoA reductase in the cholesterol biosynthetic pathway, affecting intracellular cholesterol distribution, gene expression, and proteasome activity. This leads to a reduction in Aβ production, lowering the risk of AD and demonstrating positive effects on cognitive function. Beyond their well-known lipid-lowering effects, statins may also influence AD cognitive function through mechanisms involving the gut microbiota. For instance, atorvastatin has been shown to effectively increase the abundance of intestinal *Lactobacillus* while reducing *Blautia* and *Ruminococcaceae*. This modulation of the gut-brain axis alleviates neuroinflammation and improves cognition. Both atorvastatin and rosuvastatin increase the abundance of butyrate-producing bacteria, such as *Butyricimonas*, *Bacteroides*, and *Mucispirillum*, leading to reduced IL-1β levels and improved inflammation [262]. Oral administration of simvastatin has been demonstrated to enhance gut microbial activity, increase SCFAs levels in feces, strengthen intestinal cell connections, and reduce cell death and amyloid plaque deposition in the hippocampal tissue [263]. As showed in Table 2. Furthermore, the effects and influences of statins on AD are still under ongoing exploration, with the interaction with gut microbiota being a potential mechanism.

**Table 2:** Relevant studies on the current oral drug formulations affecting the gut microbiota and lipids in AD

Category	Compound	Model	Alterations of GM	Alterations of lipids	Main effects	Reference
**Polyphenols**	Hawthorn flavonoid	D-galactose and aluminum chloride induced AD mice	*Dubosiella*↑, *Alloprevotella*↑, *Bifidobacterium*↑, *Acinetobacter*↓,	Docosapentaenoic acid↑, sphingolipid↑, PC↑	Ameliorated Aβ accumulation and abnormal activation of hippocampal microglia, improved cognitive deficits	[251]
	Curcumin	APP/PS1 mice, BV2 Microglial Cells	*Prevotellaceae*,↓ *Bacteroides*↓, *Escherichia/Shigella*↓	TREM2 expression↑	Inhibits neuroinflammation, reduce the amyloid plaque burden, improve the spatial learning and memory abilities	[252, 253]
	Bilberry anthocyanins	APP/PS1 mice	Serum and brain LPS levels↓,	SCFAs↑	Decreases hippocampal neuroinflammatory responses, Aβ plaques, reverse cognitive disfunction	[254]
	Luteolin	/	Butyrate↑, Neurotoxin cytokines↓, LPS↓,TMAO↓	SCFAs↑	Reduce neuroinflammation, intracellular tau-related neurofibrillary tangles and extracellular Aβ deposition	[264]
**Herbal medicines**	Schisandra chinensis	Aβ injection SD rats	*Lactobacillus*↑, *Firmicutes*↑, *Bacteroidetes*↑, *Pseudomonadaceae_Pseudomonas*↓,butyrate↑, propionate↑,	SCFAs↑	Improved learning and memory capacity, reduced neuroinflammation, and restoration of the integrity of the intestinal barrier	[257]
	Epimedii Folium and Curculiginis Rhizoma	LPS-induced neuroinflammatory mouse model	LPS↓	TREM2 expression↑,APOE expression↓	Inhibited neuroinflammation, ameliorated cognitive impairment	[260]
	Pyrolae herba	LPS injection C57BL6/J mice	LPS↓	TREM2 expression↓	Reduce pro-inflammatory factors, improve cognitive function	[261]
**Statin**	Atorvastatin	SD rats	*Lactobacillus*↑, *Blautia*↓, *Ruminococcaceae*↓	RA metabolism↑	Inhibit neuroinflammation, improve cognitive decline	[265]
	Simvastatin	Ovariectomized/D-galactose AD rat	Propionic acid↑, acetic acid↑, butyric acid↑, tight junctions of intestinal cells↑	SCFAs↑	Decreased cell death and Aβ plaques, reducted neuronal inflammation, protected learning and memory function	[263]

**Abbreviations**: AD = Alzheimer’s disease; APOE = Apolipoprotein E; APP = Amyloid precursor protein; Aβ = amyloid β; GM = Gut microbiota; LPS = Lipopolysaccharides; RA = Retinoic acid; SCFAs = Short-chain fatty acids; TMAO = Trimethylamine N-oxide; TREM2 = Triggering Receptor Expressed on Myeloid Cells 2

### Lifestyle

#### Dietary patterns

Dietary patterns have been shown to play a role in AD pathology. The Western diet, characterized by high fat, high protein, and low fiber intake, has been associated with a reduced abundance of beneficial microbial strains, including *Lactobacillus*, *Ruminococcaceae*, *Lachnospiraceae*, and SCFA-producing bacteria such as *Ruminococcus bromii*, *Faecalibacterium prausnitzii, Eubacterium rectale*, *Eubacterium hallii*, and *Anaerostipes coli SS2/1*, correlating with an increased risk of AD [266]. In contrast, the Mediterranean diet (MeDi), representing a balanced nutritional pattern rich in unsaturated fatty acids, vegetables, fruits, and lean meat proteins, has demonstrated the ability to effectively modulate the abundance of beneficial bacteria, including *Bifidobacterium* and *Lactobacillus*, in the gut, thereby reducing the risk of AD onset [267]. The ketogenic diet emphasizes very low carbohydrate intake and high-fat foods, exhibiting therapeutic effects in AD patients by modulating gut homeostasis, reducing neuronal overexcitation, enhancing mitochondrial metabolism, and decreasing oxidative stress [268]. In a randomized, double-blind, single-center clinical study, the Modified Mediterranean-Ketogenic Diet (MMKD), which allows increased carbohydrate consumption compared to the ketogenic diet, involves higher intake of vegetables, fruits, olive oil, as well as fats and proteins from fish sources. The results demonstrated that MMKD increased the abundance of the *Phylum Tenericutes* and the *family Enterobacteriaceae*, both negatively correlated with the expression levels of Aβ42 in the cerebrospinal fluid. This dietary approach also increased butyrate levels, restricted LPS diffusion, promoted gut barrier stability, effectively restored gut microbiota composition, enhanced steroid biosynthesis, and improved AD pathology [267]. Therefore, the modulation of gut microbiota and lipid metabolism by dietary patterns holds significant importance in the prevention of AD and the attenuation of disease progression.

#### Exercise

Exercise can stimulate the proliferation of “beneficial” microbial communities, maintaining gut microbiota balance and subsequently improving health conditions [269]. Consequently, exercise is regarded as an effective and readily available therapeutic approach, possibly the single most important and accessible lifestyle component offering protection against a broad range of diseases [270, 271]. There is a close association between exercise and the gut-brain axis. Studies have found a negative correlation between physical activity and the risk of AD, indicating that regular exercise in the elderly can prevent AD and slow cognitive decline. Therefore, exercise serves as both a preventive strategy and an intervention measure in the treatment of AD [272, 273].

A 16-week running wheel exercise regimen has been demonstrated to increase the abundance of *Firmicutes* while decreasing the abundance of *Bacteroidetes* and *Tenericutes*, effectively improving gut microbiota composition and memory [274]. Running exercise has also been shown to increase the microbial content of *Eubacteria*, *Roseburia*, and *Clostridia* in the intestines of APP/PS1 mice, while decreasing the abundance of *Prevotella*, *Bacteroides*, *Bacteroides fragilis*, and *L. johnsonii*. This alteration inhibits the transfer of LPS to the brain, thereby alleviating LPS-induced neuroinflammation and improving cognitive function and pathological markers in AD mice [273, 275]. Moreover, voluntary wheel running (VWR) exercise has been found to upregulate the abundance of phylum *Bacteroidetes* and genus *Prevotella* while reducing the abundance of phylum *Actinobacteria* and *TM7*, as well as genus *Oscillospira* and *Ruminococcus*. This modulation mitigates cognitive dysfunction induced by TMAO [276]. These studies collectively indicate that exercise serves as an effective measure in regulating gut microbiota to improve the pathological progression of AD, presenting substantial potential in both the treatment and prevention of AD. However, it is imperative to recognize that as an intervention for AD, further research is needed to explore the specifics of exercise protocols, modes, and intensities.

## Conclusions and perspectives

As emphasized in this review, the intricate interplay between gut microbiota and lipid metabolism in the pathogenesis of AD is a noteworthy research area. We summarize the current research evidence, highlighting the central role of gut microbiota-derived metabolites such as SCFAs, LPS, TMAO, BAs, and tryptophan indole derivatives in the lipid metabolism disruption of AD pathology. As the ultimate products of gut microbiota, microbial metabolites not only interact with key lipid metabolism genes such as APOE, TREM2, ABCA1, ABCA7, SREBP1, SREBP2, and CLU but also participate in AD lipid metabolism and pathological processes by regulating Aβ and tau pathologies, neuroinflammation, oxidative stress, mitochondrial dysfunction, and epigenetic regulation. These mechanisms are interconnected and mutually influential, and while we have only begun to elucidate their complex pathological associations, the exact underlying mechanisms warrant further in-depth exploration. In future research, the application of omics techniques such as metagenomics, meta transcriptomics, and metabolomics will aid in uncovering the intricate mechanisms governing the relationship between lipids, gut microbiota, and AD, providing a deeper understanding of their interconnections. Moreover, current therapeutic strategies and drugs for AD remain limited, and the close connection between gut microbiota and lipid metabolism provides new insights into treatment approaches. Supplementation with potential beneficial bacteria through probiotics, prebiotics, and fecal microbiota transplantation may impede or slow the pathological progression of AD. Additionally, the direct impact of diet on the production of microbial metabolites should not be overlooked, emphasizing the importance of dietary regulation. Exercise, as a beneficial lifestyle factor, also holds importance in the prevention of Alzheimer’s disease. Polyphenols, herbal medicines, and statin drugs demonstrate neuroprotective effects in the gut microbiota and lipid metabolism of AD, potentially holding translational value.

### Electronic supplementary material

Below is the link to the electronic supplementary material.

Supplementary Material 1

## Data Availability

Not applicable.

## Abbreviations

25HC: 25-hydroxycholesterol
AOF: Alpinae Oxyphyllae Fructus
AD: Alzheimer’s disease
Aβ: Amyloid beta
APP: Amyloid precursor protein
APOE: polipoprotein E
AA: Arachidonic acid
AhR: Aryl hydrocarbon receptor
ABCA1: ATP-binding cassette A1
ABCA7: ATP-binding cassette A7
ABCA: ATP-binding cassette subfamily A
BAs: Bile acids
BBB: Blood-brain barrier
CRL: Candida rugosa lipase
Cer: Ceramide
CSF: Cerebrospinal fluid
CDCA: Chenodeoxycholic acid
CHOL: Cholesterol
ACAT: Cholesterol acyltransferase
CE: Cholesterol ester
CH25H: Cholesterol-25-hydroxylase
CA: Cholic acid
UDCA: Cholic ursodeoxycholic acid
CLU: Clusterin
DCA: Deoxycholic acid
DHA: Docosahexaenoic acid
EPA: Eicosapentaenoic acid
PlsEtns: Ethanolamine plasmalogens
ePtdSer: Externalized phosphatidylserine
FXR: Farnesoid X receptor
FAs: Fatty acids
FMT: Fecal microbiota transplantation
FFA: Free fatty acid
GPBAR1/TGR5: G protein-coupled bile acid receptor 1/Takeda G protein-coupled receptor 5
GPRs: G protein-coupled receptors
GWAS: Genome-wide association studies
GSK3β: Glycogen synthase kinase 3 beta
GM: Gut microbiota
GPCRs: G-protein coupled receptors
GF: Germ-free
HF: Hawthorn flavonoid
HATs: Histone acetyltransferases
HDACs: Histone deacetylases
IPAM: Indole-3-propionamide
IPA: Indole-3-propionic acid
IL-1β: Interleukin-1 beta
LOAD: Late-onset Alzheimer’s disease
LRP1: LDLR-related protein 1
LPS: Lipopolysaccharides
LCA: Lithocholic acid
LDLR: Low-density lipoprotein receptor
LRP-1: Low-density lipoprotein receptor-related protein 1
MCI: Mild Cognitive Impairment
MMKD: Modified Mediterranean-Ketogenic Diet
CML: N6-carboxymethyllysine
NDAN: Nondemented individuals with AD neuropathology
NFTs: Neurofibrillary tangles
NLRP3: NOD-like receptor protein 3
NF-κB: Nuclear factor kappa B
PA: Palmitic acid
PGE2: Prostaglandin E2
PGF1α: Prostaglandin f1alpha
PGF2α: Prostaglandin F2alpha
PC: Phosphatidylcholine
PE: Phosphatidylethanolamine
PUFAs: Polyunsaturated fatty acids
PtdSer: Phosphatidylserine
RA: Retinoic acid
RNS: Reactive nitrogen species
ROS: Reactive oxygen species
SFAs: Saturated fatty acids
SCFAs: Short-chain fatty acids
S1P: Sphingosine-1-phosphate
SPF: Specific pathogen-free
SREBPs: Sterol regulatory-element binding proteins
TUDCA: Tauroursodeoxycholic acid
TLRs: Toll-like receptors
TLR4: Toll-like receptor 4
TCA: Tricarboxylic acid
TREM2: Triggering Receptor Expressed on Myeloid Cells 2
TMA: Trimethylamine
TMAO: Trimethylamine N-oxide
TRP: Tryptophan
TRYCATs: Tryptophan catabolites
TNFα: Tumor necrosis factor alpha
UFAs: Unsaturated fatty acids
BACE1: β-site amyloid precursor protein-cleaving enzyme 1
ω-3 PUFAs: ω-3 polyunsaturated fatty acids
ω-6 PUFAs: ω-6 polyunsaturated fatty acids

## References

[1] Long JM, Holtzman DM (2019). Alzheimer Disease: an update on pathobiology and treatment strategies. Cell.

[2] Scheltens P, De Strooper B, Kivipelto M, Holstege H, Chetelat G, Teunissen CE (2021). Alzheimer’s disease. Lancet.

[3] Knopman DS, Amieva H, Petersen RC, Chetelat G, Holtzman DM, Hyman BT (2021). Alzheimer disease. Nat Rev Dis Primers.

[4] Graff-Radford J, Yong KXX, Apostolova LG, Bouwman FH, Carrillo M, Dickerson BC (2021). New insights into atypical Alzheimer’s disease in the era of biomarkers. Lancet Neurol.

[5] Di Paolo G, Kim TW (2011). Linking lipids to Alzheimer’s disease: cholesterol and beyond. Nat Rev Neurosci.

[6] Sastry PS (1985). Lipids of nervous tissue: composition and metabolism. Prog Lipid Res.

[7] Schonfeld P, Reiser G (2013). Why does brain metabolism not favor burning of fatty acids to provide energy? Reflections on disadvantages of the use of free fatty acids as fuel for brain. J Cereb Blood Flow Metab.

[8] Sezgin E, Levental I, Mayor S, Eggeling C (2017). The mystery of membrane organization: composition, regulation and roles of lipid rafts. Nat Rev Mol Cell Biol.

[9] Yoon JH, Seo Y, Jo YS, Lee S, Cho E, Cazenave-Gassiot A (2022). Brain lipidomics: from functional landscape to clinical significance. Sci Adv.

[10] Sebastiao AM, Colino-Oliveira M, Assaife-Lopes N, Dias RB, Ribeiro JA (2013). Lipid rafts, synaptic transmission and plasticity: impact in age-related neurodegenerative diseases. Neuropharmacology.

[11] Kao YC, Ho PC, Tu YK, Jou IM, Tsai KJ. Lipids and Alzheimer’s Disease. Int J Mol Sci. 2020;21(4). 10.3390/ijms21041505 PMC7073164 32098382

[12] Cardoso S, Carvalho C, Correia SC, Seica RM, Moreira PI (2016). Alzheimer’s Disease: from mitochondrial perturbations to mitochondrial medicine. Brain Pathol.

[13] Yin F, Sancheti H, Patil I, Cadenas E (2016). Energy metabolism and inflammation in brain aging and Alzheimer’s disease. Free Radic Biol Med.

[14] Hamilton LK, Dufresne M, Joppe SE, Petryszyn S, Aumont A, Calon F (2015). Aberrant lipid metabolism in the Forebrain Niche suppresses adult neural stem cell proliferation in an animal model of Alzheimer’s Disease. Cell Stem Cell.

[15] Ferre-Gonzalez L, Lloret A, Chafer-Pericas C (2023). Systematic review of brain and blood lipidomics in Alzheimer’s disease mouse models. Prog Lipid Res.

[16] Teitsdottir UD, Halldorsson S, Rolfsson O, Lund SH, Jonsdottir MK, Snaedal J (2021). Cerebrospinal fluid C18 Ceramide Associates with markers of Alzheimer’s disease and inflammation at the pre- and early stages of Dementia. J Alzheimers Dis.

[17] Mielke MM, Bandaru VV, Haughey NJ, Xia J, Fried LP, Yasar S (2012). Serum ceramides increase the risk of Alzheimer disease: the women’s Health and Aging Study II. Neurology.

[18] Valdes AM, Walter J, Segal E, Spector TD (2018). Role of the gut microbiota in nutrition and health. BMJ.

[19] Cryan JF, O’Riordan KJ, Cowan CSM, Sandhu KV, Bastiaanssen TFS, Boehme M (2019). The Microbiota-Gut-Brain Axis. Physiol Rev.

[20] Martin CR, Osadchiy V, Kalani A, Mayer EA (2018). The brain-gut-Microbiome Axis. Cell Mol Gastroenterol Hepatol.

[21] Fan Y, Pedersen O (2021). Gut microbiota in human metabolic health and disease. Nat Rev Microbiol.

[22] Lynch SV, Pedersen O (2016). The human intestinal microbiome in Health and Disease. N Engl J Med.

[23] Long-Smith C, O’Riordan KJ, Clarke G, Stanton C, Dinan TG, Cryan JF (2020). Microbiota-Gut-Brain Axis: New Therapeutic opportunities. Annu Rev Pharmacol Toxicol.

[24] Fulling C, Dinan TG, Cryan JF (2019). Gut microbe to Brain Signaling: what happens in Vagus. Neuron.

[25] Sun BL, Li WW, Wang J, Xu YL, Sun HL, Tian DY (2019). Gut microbiota alteration and its time course in a Tauopathy Mouse Model. J Alzheimers Dis.

[26] Zhuang ZQ, Shen LL, Li WW, Fu X, Zeng F, Gui L (2018). Gut microbiota is altered in patients with Alzheimer’s Disease. J Alzheimers Dis.

[27] Cattaneo A, Cattane N, Galluzzi S, Provasi S, Lopizzo N, Festari C (2017). Association of brain amyloidosis with pro-inflammatory gut bacterial taxa and peripheral inflammation markers in cognitively impaired elderly. Neurobiol Aging.

[28] Westfall S, Lomis N, Kahouli I, Dia SY, Singh SP, Prakash S (2017). Microbiome, probiotics and neurodegenerative diseases: deciphering the gut brain axis. Cell Mol Life Sci.

[29] Vogt NM, Kerby RL, Dill-McFarland KA, Harding SJ, Merluzzi AP, Johnson SC (2017). Gut microbiome alterations in Alzheimer’s disease. Sci Rep.

[30] Liu P, Wu L, Peng G, Han Y, Tang R, Ge J (2019). Altered microbiomes distinguish Alzheimer’s disease from amnestic mild cognitive impairment and health in a Chinese cohort. Brain Behav Immun.

[31] Ferreiro AL, Choi J, Ryou J, Newcomer EP, Thompson R, Bollinger RM (2023). Gut microbiome composition may be an indicator of preclinical Alzheimer’s disease. Sci Transl Med.

[32] Wang YR, Liang CR, Heng T, Zhang T, Hu XT, Long Y (2023). Circulating antibodies to Helicobacter pylori are associated with biomarkers of neurodegeneration in cognitively intact adults. Asian J Psychiatr.

[33] Shen L, Liu L, Ji HF (2017). Alzheimer’s disease histological and behavioral manifestations in transgenic mice correlate with specific gut Microbiome State. J Alzheimers Dis.

[34] Brandscheid C, Schuck F, Reinhardt S, Schafer KH, Pietrzik CU, Grimm M (2017). Altered gut Microbiome Composition and Tryptic Activity of the 5xFAD Alzheimer’s mouse model. J Alzheimers Dis.

[35] Chandra S, Sisodia SS, Vassar RJ (2023). The gut microbiome in Alzheimer’s disease: what we know and what remains to be explored. Mol Neurodegener.

[36] Mezo C, Dokalis N, Mossad O, Staszewski O, Neuber J, Yilmaz B (2020). Different effects of constitutive and induced microbiota modulation on microglia in a mouse model of Alzheimer’s disease. Acta Neuropathol Commun.

[37] Minter MR, Zhang C, Leone V, Ringus DL, Zhang X, Oyler-Castrillo P (2016). Antibiotic-induced perturbations in gut microbial diversity influences neuro-inflammation and amyloidosis in a murine model of Alzheimer’s disease. Sci Rep.

[38] Harach T, Marungruang N, Duthilleul N, Cheatham V, Mc Coy KD, Frisoni G (2017). Reduction of Abeta amyloid pathology in APPPS1 transgenic mice in the absence of gut microbiota. Sci Rep.

[39] Zhang Y, Shen Y, Liufu N, Liu L, Li W, Shi Z (2023). Transmission of Alzheimer’s disease-associated microbiota dysbiosis and its impact on cognitive function: evidence from mice and patients. Mol Psychiatry.

[40] Kim MS, Kim Y, Choi H, Kim W, Park S, Lee D (2020). Transfer of a healthy microbiota reduces amyloid and tau pathology in an Alzheimer’s disease animal model. Gut.

[41] Simao DO, Vieira VS, Tosatti JAG, Gomes KB, Lipids. Gut Microbiota, and the Complex Relationship with Alzheimer’s Disease: A Narrative Review. Nutrients. 2023;15(21). 10.3390/nu15214661 PMC10649859 37960314

[42] Cheng X, Tan Y, Li H, Huang J, Zhao D, Zhang Z (2022). Fecal 16S rRNA sequencing and multi-compartment metabolomics revealed gut microbiota and metabolites interactions in APP/PS1 mice. Comput Biol Med.

[43] Qian X, Hai W, Chen S, Zhang M, Jiang X, Tang H (2023). Multi-omics data reveals aberrant gut microbiota-host glycerophospholipid metabolism in association with neuroinflammation in APP/PS1 mice. Gut Microbes.

[44] Mirzaei R, Bouzari B, Hosseini-Fard SR, Mazaheri M, Ahmadyousefi Y, Abdi M (2021). Role of microbiota-derived short-chain fatty acids in nervous system disorders. Biomed Pharmacother.

[45] Chen C, Liao J, Xia Y, Liu X, Jones R, Haran J (2022). Gut microbiota regulate Alzheimer’s disease pathologies and cognitive disorders via PUFA-associated neuroinflammation. Gut.

[46] Bonfili L, Cuccioloni M, Gong C, Cecarini V, Spina M, Zheng Y (2022). Gut microbiota modulation in Alzheimer’s disease: focus on lipid metabolism. Clin Nutr.

[47] Lei E, Vacy K, Boon WC (2016). Fatty acids and their therapeutic potential in neurological disorders. Neurochem Int.

[48] Li X, Bi X, Wang S, Zhang Z, Li F, Zhao AZ (2019). Therapeutic potential of omega-3 polyunsaturated fatty acids in Human Autoimmune diseases. Front Immunol.

[49] Snowden SG, Ebshiana AA, Hye A, An Y, Pletnikova O, O’Brien R (2017). Association between fatty acid metabolism in the brain and Alzheimer disease neuropathology and cognitive performance: a nontargeted metabolomic study. PLoS Med.

[50] Andrieu S, Guyonnet S, Coley N, Cantet C, Bonnefoy M, Bordes S (2017). Effect of long-term omega 3 polyunsaturated fatty acid supplementation with or without multidomain intervention on cognitive function in elderly adults with memory complaints (MAPT): a randomised, placebo-controlled trial. Lancet Neurol.

[51] El Shatshat A, Pham AT, Rao PPN (2019). Interactions of polyunsaturated fatty acids with amyloid peptides Abeta40 and Abeta42. Arch Biochem Biophys.

[52] Cunnane SC, Schneider JA, Tangney C, Tremblay-Mercier J, Fortier M, Bennett DA (2012). Plasma and brain fatty acid profiles in mild cognitive impairment and Alzheimer’s disease. J Alzheimers Dis.

[53] Bogie JFJ, Haidar M, Kooij G, Hendriks JJA (2020). Fatty acid metabolism in the progression and resolution of CNS disorders. Adv Drug Deliv Rev.

[54] Yamashima T, Ota T, Mizukoshi E, Nakamura H, Yamamoto Y, Kikuchi M (2020). Intake of omega-6 polyunsaturated fatty acid-rich vegetable oils and risk of Lifestyle diseases. Adv Nutr.

[55] Simopoulos AP (2002). The importance of the ratio of omega-6/omega-3 essential fatty acids. Biomed Pharmacother.

[56] Gustafson DR, Backman K, Scarmeas N, Stern Y, Manly JJ, Mayeux R (2020). Dietary fatty acids and risk of Alzheimer’s disease and related dementias: observations from the Washington Heights-Hamilton Heights-Inwood Columbia Aging Project (WHICAP). Alzheimers Dement.

[57] Howe AM, Burke S, O’Reilly ME, McGillicuddy FC, Costello DA (2022). Palmitic acid and oleic acid differently modulate TLR2-Mediated inflammatory responses in Microglia and macrophages. Mol Neurobiol.

[58] Fraser T, Tayler H, Love S (2010). Fatty acid composition of frontal, temporal and parietal neocortex in the normal human brain and in Alzheimer’s disease. Neurochem Res.

[59] Flores-Leon M, Perez-Dominguez M, Gonzalez-Barrios R, Arias C (2019). Palmitic Acid-Induced NAD(+) depletion is Associated with the reduced function of SIRT1 and increased expression of BACE1 in hippocampal neurons. Neurochem Res.

[60] Marwarha G, Claycombe-Larson K, Lund J, Ghribi O (2019). Palmitate-Induced SREBP1 expression and activation underlies the increased BACE 1 activity and amyloid Beta Genesis. Mol Neurobiol.

[61] Chan RB, Oliveira TG, Cortes EP, Honig LS, Duff KE, Small SA (2012). Comparative lipidomic analysis of mouse and human brain with Alzheimer disease. J Biol Chem.

[62] Diaz G, Lengele L, Sourdet S, Soriano G, de Souto Barreto P (2022). Nutrients and amyloid beta status in the brain: a narrative review. Ageing Res Rev.

[63] Umeda T, Tomiyama T, Kitajima E, Idomoto T, Nomura S, Lambert MP (2012). Hypercholesterolemia accelerates intraneuronal accumulation of Abeta oligomers resulting in memory impairment in Alzheimer’s disease model mice. Life Sci.

[64] Bossaerts L, Cacace R, Van Broeckhoven C (2022). The role of ATP-binding cassette subfamily A in the etiology of Alzheimer’s disease. Mol Neurodegener.

[65] Wood WG, Li L, Muller WE, Eckert GP (2014). Cholesterol as a causative factor in Alzheimer’s disease: a debatable hypothesis. J Neurochem.

[66] Silva T, Teixeira J, Remiao F, Borges F (2013). Alzheimer’s disease, cholesterol, and statins: the junctions of important metabolic pathways. Angew Chem Int Ed Engl.

[67] van der Kant R, Langness VF, Herrera CM, Williams DA, Fong LK, Leestemaker Y (2019). Cholesterol metabolism is a Druggable Axis that independently regulates tau and amyloid-beta in iPSC-Derived Alzheimer’s disease neurons. Cell Stem Cell.

[68] Ooi KM, Vacy K, Boon WC (2021). Fatty acids and beyond: Age and Alzheimer’s disease related changes in lipids reveal the neuro-nutraceutical potential of lipids in cognition. Neurochem Int.

[69] Gonzalez-Dominguez R, Garcia-Barrera T, Gomez-Ariza JL (2014). Combination of metabolomic and phospholipid-profiling approaches for the study of Alzheimer’s disease. J Proteom.

[70] Blusztajn JK, Slack BE (2023). Accelerated breakdown of Phosphatidylcholine and Phosphatidylethanolamine is a predominant brain metabolic defect in Alzheimer’s Disease. J Alzheimers Dis.

[71] Varma VR, Oommen AM, Varma S, Casanova R, An Y, Andrews RM (2018). Brain and blood metabolite signatures of pathology and progression in Alzheimer disease: a targeted metabolomics study. PLoS Med.

[72] Rodriguez-Cuenca S, Pellegrinelli V, Campbell M, Oresic M, Vidal-Puig A (2017). Sphingolipids and glycerophospholipids - the Ying and Yang of lipotoxicity in metabolic diseases. Prog Lipid Res.

[73] Wood PL, Mankidy R, Ritchie S, Heath D, Wood JA, Flax J (2010). Circulating plasmalogen levels and Alzheimer Disease Assessment Scale-cognitive scores in Alzheimer patients. J Psychiatry Neurosci.

[74] Su XQ, Wang J, Sinclair AJ (2019). Plasmalogens and Alzheimer’s disease: a review. Lipids Health Dis.

[75] Dorninger F, Forss-Petter S, Berger J (2017). From peroxisomal disorders to common neurodegenerative diseases - the role of ether phospholipids in the nervous system. FEBS Lett.

[76] Svennerholm L (1968). Distribution and fatty acid composition of phosphoglycerides in normal human brain. J Lipid Res.

[77] Bader Lange ML, Cenini G, Piroddi M, Abdul HM, Sultana R, Galli F (2008). Loss of phospholipid asymmetry and elevated brain apoptotic protein levels in subjects with amnestic mild cognitive impairment and Alzheimer disease. Neurobiol Dis.

[78] Kim HY, Huang BX, Spector AA (2014). Phosphatidylserine in the brain: metabolism and function. Prog Lipid Res.

[79] Wang Y, Cella M, Mallinson K, Ulrich JD, Young KL, Robinette ML (2015). TREM2 lipid sensing sustains the microglial response in an Alzheimer’s disease model. Cell.

[80] Scott-Hewitt N, Perrucci F, Morini R, Erreni M, Mahoney M, Witkowska A (2020). Local externalization of phosphatidylserine mediates developmental synaptic pruning by microglia. EMBO J.

[81] Popescu AS, Butler CA, Allendorf DH, Piers TM, Mallach A, Roewe J (2023). Alzheimer’s disease-associated R47H TREM2 increases, but wild-type TREM2 decreases, microglial phagocytosis of synaptosomes and neuronal loss. Glia.

[82] Fracassi A, Marcatti M, Tumurbaatar B, Woltjer R, Moreno S, Taglialatela G (2023). TREM2-induced activation of microglia contributes to synaptic integrity in cognitively intact aged individuals with Alzheimer’s neuropathology. Brain Pathol.

[83] Rueda-Carrasco J, Sokolova D, Lee SE, Childs T, Jurcakova N, Crowley G (2023). Microglia-synapse engulfment via PtdSer-TREM2 ameliorates neuronal hyperactivity in Alzheimer’s disease models. EMBO J.

[84] Jesko H, Stepien A, Lukiw WJ, Strosznajder RP (2019). The Cross-talk between sphingolipids and insulin-like growth factor signaling: significance for aging and neurodegeneration. Mol Neurobiol.

[85] van Echten-Deckert G, Walter J (2012). Sphingolipids: critical players in Alzheimer’s disease. Prog Lipid Res.

[86] Xu J, Bankov G, Kim M, Wretlind A, Lord J, Green R (2020). Integrated lipidomics and proteomics network analysis highlights lipid and immunity pathways associated with Alzheimer’s disease. Transl Neurodegener.

[87] Crivelli SM, Giovagnoni C, Visseren L, Scheithauer AL, de Wit N, den Hoedt S (2020). Sphingolipids in Alzheimer’s disease, how can we target them?. Adv Drug Deliv Rev.

[88] Parveen F, Bender D, Law SH, Mishra VK, Chen CC, Ke LY. Role of ceramidases in Sphingolipid Metabolism and Human diseases. Cells. 2019;8(12). 10.3390/cells8121573 PMC6952831 31817238

[89] Dinkins MB, Enasko J, Hernandez C, Wang G, Kong J, Helwa I (2016). Neutral Sphingomyelinase-2 Deficiency ameliorates Alzheimer’s Disease Pathology and improves cognition in the 5XFAD mouse. J Neurosci.

[90] Krautkramer KA, Fan J, Backhed F (2021). Gut microbial metabolites as multi-kingdom intermediates. Nat Rev Microbiol.

[91] Wu L, Han Y, Zheng Z, Peng G, Liu P, Yue S et al. Altered Gut Microbial Metabolites in Amnestic Mild Cognitive Impairment and Alzheimer’s Disease: Signals in Host-Microbe Interplay. Nutrients. 2021;13(1). 10.3390/nu13010228 PMC7829997 33466861

[92] van der Hee B, Wells JM (2021). Microbial regulation of host physiology by short-chain fatty acids. Trends Microbiol.

[93] Qian XH, Xie RY, Liu XL, Chen SD, Tang HD (2022). Mechanisms of short-chain fatty acids derived from Gut Microbiota in Alzheimer’s Disease. Aging Dis.

[94] Liu J, Li H, Gong T, Chen W, Mao S, Kong Y (2020). Anti-neuroinflammatory effect of short-chain fatty acid acetate against Alzheimer’s Disease via Upregulating GPR41 and inhibiting ERK/JNK/NF-kappaB. J Agric Food Chem.

[95] Hoyles L, Snelling T, Umlai UK, Nicholson JK, Carding SR, Glen RC (2018). Microbiome-host systems interactions: protective effects of propionate upon the blood-brain barrier. Microbiome.

[96] Govindarajan N, Agis-Balboa RC, Walter J, Sananbenesi F, Fischer A (2011). Sodium butyrate improves memory function in an Alzheimer’s disease mouse model when administered at an advanced stage of disease progression. J Alzheimers Dis.

[97] Ge X, Zheng M, Hu M, Fang X, Geng D, Liu S et al. Butyrate ameliorates quinolinic acid-induced cognitive decline in obesity models. J Clin Invest. 2023;133(4). 10.1172/JCI154612 PMC9927952 36787221

[98] Colombo AV, Sadler RK, Llovera G, Singh V, Roth S, Heindl S et al. Microbiota-derived short chain fatty acids modulate microglia and promote abeta plaque deposition. Elife. 2021;10. 10.7554/eLife.59826 PMC8043748 33845942

[99] Erny D, Dokalis N, Mezo C, Castoldi A, Mossad O, Staszewski O (2021). Microbiota-derived acetate enables the metabolic fitness of the brain innate immune system during health and disease. Cell Metab.

[100] Seo DO, O’Donnell D, Jain N, Ulrich JD, Herz J, Li Y (2023). ApoE isoform- and microbiota-dependent progression of neurodegeneration in a mouse model of tauopathy. Science.

[101] Zhou Y, Xie L, Schroder J, Schuster IS, Nakai M, Sun G (2023). Dietary Fiber and Microbiota Metabolite Receptors Enhance Cognition and alleviate Disease in the 5xFAD mouse model of Alzheimer’s Disease. J Neurosci.

[102] Spichak S, Bastiaanssen TFS, Berding K, Vlckova K, Clarke G, Dinan TG (2021). Mining microbes for mental health: determining the role of microbial metabolic pathways in human brain health and disease. Neurosci Biobehav Rev.

[103] Connell E, Le Gall G, Pontifex MG, Sami S, Cryan JF, Clarke G (2022). Microbial-derived metabolites as a risk factor of age-related cognitive decline and dementia. Mol Neurodegener.

[104] MahmoudianDehkordi S, Arnold M, Nho K, Ahmad S, Jia W, Xie G (2019). Altered bile acid profile associates with cognitive impairment in Alzheimer’s disease-An emerging role for gut microbiome. Alzheimers Dement.

[105] Baloni P, Funk CC, Yan J, Yurkovich JT, Kueider-Paisley A, Nho K (2020). Metabolic Network Analysis reveals altered bile acid synthesis and metabolism in Alzheimer’s Disease. Cell Rep Med.

[106] Huang F, Pariante CM, Borsini A (2022). From dried bear bile to molecular investigation: a systematic review of the effect of bile acids on cell apoptosis, oxidative stress and inflammation in the brain, across pre-clinical models of neurological, neurodegenerative and neuropsychiatric disorders. Brain Behav Immun.

[107] Dionisio PA, Amaral JD, Ribeiro MF, Lo AC, D’Hooge R, Rodrigues CM (2015). Amyloid-beta pathology is attenuated by tauroursodeoxycholic acid treatment in APP/PS1 mice after disease onset. Neurobiol Aging.

[108] Yanguas-Casas N, Barreda-Manso MA, Nieto-Sampedro M, Romero-Ramirez L (2017). TUDCA: an agonist of the bile acid receptor GPBAR1/TGR5 with anti-inflammatory effects in Microglial cells. J Cell Physiol.

[109] Zangerolamo L, Vettorazzi JF, Rosa LRO, Carneiro EM, Barbosa HCL (2021). The bile acid TUDCA and neurodegenerative disorders: an overview. Life Sci.

[110] Khalaf K, Tornese P, Cocco A, Albanese A (2022). Tauroursodeoxycholic acid: a potential therapeutic tool in neurodegenerative diseases. Transl Neurodegener.

[111] Zhan X, Stamova B, Jin LW, DeCarli C, Phinney B, Sharp FR (2016). Gram-negative bacterial molecules associate with Alzheimer disease pathology. Neurology.

[112] Zhao Y, Cong L, Jaber V, Lukiw WJ (2017). Microbiome-Derived Lipopolysaccharide Enriched in the Perinuclear Region of Alzheimer’s Disease Brain. Front Immunol.

[113] Kim HS, Kim S, Shin SJ, Park YH, Nam Y, Kim CW (2021). Gram-negative bacteria and their lipopolysaccharides in Alzheimer’s disease: pathologic roles and therapeutic implications. Transl Neurodegener.

[114] Brown GC (2019). The endotoxin hypothesis of neurodegeneration. J Neuroinflammation.

[115] Wu Z, Ni J, Liu Y, Teeling JL, Takayama F, Collcutt A (2017). Cathepsin B plays a critical role in inducing Alzheimer’s disease-like phenotypes following chronic systemic exposure to lipopolysaccharide from Porphyromonas gingivalis in mice. Brain Behav Immun.

[116] Erickson MA, Hartvigson PE, Morofuji Y, Owen JB, Butterfield DA, Banks WA (2012). Lipopolysaccharide impairs amyloid beta efflux from brain: altered vascular sequestration, cerebrospinal fluid reabsorption, peripheral clearance and transporter function at the blood-brain barrier. J Neuroinflammation.

[117] Ye X, Zhu M, Che X, Wang H, Liang XJ, Wu C (2020). Lipopolysaccharide induces neuroinflammation in microglia by activating the MTOR pathway and downregulating Vps34 to inhibit autophagosome formation. J Neuroinflammation.

[118] Yao C, Liu X, Tang Y, Wang C, Duan C, Liu X (2023). Lipopolysaccharide induces inflammatory microglial activation through CD147-mediated matrix metalloproteinase expression. Environ Sci Pollut Res Int.

[119] Calvo-Rodriguez M, Garcia-Rodriguez C, Villalobos C, Nunez L (2020). Role of toll like receptor 4 in Alzheimer’s Disease. Front Immunol.

[120] Miron J, Picard C, Frappier J, Dea D, Theroux L, Poirier J (2018). TLR4 gene expression and pro-inflammatory cytokines in Alzheimer’s Disease and in response to hippocampal deafferentation in rodents. J Alzheimers Dis.

[121] Kim S, Chung H, Ngoc Mai H, Nam Y, Shin SJ, Park YH et al. Low-Dose Ionizing Radiation Modulates Microglia Phenotypes in the Models of Alzheimer’s Disease. Int J Mol Sci. 2020;21(12). 10.3390/ijms21124532 PMC7353052 32630597

[122] Izumi Y, Cashikar AG, Krishnan K, Paul SM, Covey DF, Mennerick SJ (2021). A proinflammatory stimulus disrupts hippocampal plasticity and learning via Microglial activation and 25-Hydroxycholesterol. J Neurosci.

[123] Wong MY, Lewis M, Doherty JJ, Shi Y, Cashikar AG, Amelianchik A (2020). 25-Hydroxycholesterol amplifies microglial IL-1beta production in an apoE isoform-dependent manner. J Neuroinflammation.

[124] Cashikar AG, Toral-Rios D, Timm D, Romero J, Strickland M, Long JM (2023). Regulation of astrocyte lipid metabolism and ApoE secretionby the microglial oxysterol, 25-hydroxycholesterol. J Lipid Res.

[125] Zhang Y, Wang Y, Ke B, Du J (2021). TMAO: how gut microbiota contributes to heart failure. Transl Res.

[126] Vogt NM, Romano KA, Darst BF, Engelman CD, Johnson SC, Carlsson CM (2018). The gut microbiota-derived metabolite trimethylamine N-oxide is elevated in Alzheimer’s disease. Alzheimers Res Ther.

[127] Chen ML, Zhu XH, Ran L, Lang HD, Yi L, Mi MT. Trimethylamine-N-Oxide induces vascular inflammation by activating the NLRP3 Inflammasome through the SIRT3-SOD2-mtROS signaling pathway. J Am Heart Assoc. 2017;6(9). 10.1161/JAHA.117.006347 PMC5634285 28871042

[128] Wilson A, McLean C, Kim RB (2016). Trimethylamine-N-oxide: a link between the gut microbiome, bile acid metabolism, and atherosclerosis. Curr Opin Lipidol.

[129] Li D, Ke Y, Zhan R, Liu C, Zhao M, Zeng A (2018). Trimethylamine-N-oxide promotes brain aging and cognitive impairment in mice. Aging Cell.

[130] Gao Q, Wang Y, Wang X, Fu S, Zhang X, Wang RT (2019). Decreased levels of circulating trimethylamine N-oxide alleviate cognitive and pathological deterioration in transgenic mice: a potential therapeutic approach for Alzheimer’s disease. Aging.

[131] Li D, Yu S, Long Y, Shi A, Deng J, Ma Y (2022). Tryptophan metabolism: mechanism-oriented therapy for neurological and psychiatric disorders. Front Immunol.

[132] Agus A, Planchais J, Sokol H (2018). Gut microbiota regulation of Tryptophan Metabolism in Health and Disease. Cell Host Microbe.

[133] Salminen A (2023). Activation of aryl hydrocarbon receptor (AhR) in Alzheimer’s disease: role of tryptophan metabolites generated by gut host-microbiota. J Mol Med (Berl).

[134] Ramprasath T, Han YM, Zhang D, Yu CJ, Zou MH (2021). Tryptophan catabolism and inflammation: a Novel Therapeutic Target for aortic diseases. Front Immunol.

[135] Wang HC, Wong TH, Wang LT, Su HH, Yu HY, Wu AH (2019). Aryl hydrocarbon receptor signaling promotes ORMDL3-dependent generation of sphingosine-1-phosphate by inhibiting sphingosine-1-phosphate lyase. Cell Mol Immunol.

[136] Majumder S, Kono M, Lee YT, Byrnes C, Li C, Tuymetova G (2020). A genome-wide CRISPR/Cas9 screen reveals that the aryl hydrocarbon receptor stimulates sphingolipid levels. J Biol Chem.

[137] Pappolla MA, Perry G, Fang X, Zagorski M, Sambamurti K, Poeggeler B (2021). Indoles as essential mediators in the gut-brain axis. Their role in Alzheimer’s disease. Neurobiol Dis.

[138] Sun J, Zhang Y, Kong Y, Ye T, Yu Q, Kumaran Satyanarayanan S (2022). Microbiota-derived metabolite indoles induced aryl hydrocarbon receptor activation and inhibited neuroinflammation in APP/PS1 mice. Brain Behav Immun.

[139] George N, Jawaid Akhtar M, Al Balushi KA, Alam Khan S (2022). Rational drug design strategies for the development of promising multi-target directed indole hybrids as Anti-alzheimer agents. Bioorg Chem.

[140] Chen YC, Chiu YJ, Lin CH, Hsu WC, Wu JL, Huang CH (2019). Indole Compound NC009-1 augments APOE and TRKA in Alzheimer’s Disease Cell and Mouse models for Neuroprotection and Cognitive Improvement. J Alzheimers Dis.

[141] van der Velpen V, Teav T, Gallart-Ayala H, Mehl F, Konz I, Clark C (2019). Systemic and central nervous system metabolic alterations in Alzheimer’s disease. Alzheimers Res Ther.

[142] Kunkle BW, Grenier-Boley B, Sims R, Bis JC, Damotte V, Naj AC (2019). Genetic meta-analysis of diagnosed Alzheimer’s disease identifies new risk loci and implicates Abeta, tau, immunity and lipid processing. Nat Genet.

[143] Karch CM, Goate AM (2015). Alzheimer’s disease risk genes and mechanisms of disease pathogenesis. Biol Psychiatry.

[144] Bellenguez C, Kucukali F, Jansen IE, Kleineidam L, Moreno-Grau S, Amin N (2022). New insights into the genetic etiology of Alzheimer’s disease and related dementias. Nat Genet.

[145] Picard C, Julien C, Frappier J, Miron J, Theroux L, Dea D (2018). Alterations in cholesterol metabolism-related genes in sporadic Alzheimer’s disease. Neurobiol Aging.

[146] Serrano-Pozo A, Das S, Hyman BT (2021). APOE and Alzheimer’s disease: advances in genetics, pathophysiology, and therapeutic approaches. Lancet Neurol.

[147] Raulin AC, Doss SV, Trottier ZA, Ikezu TC, Bu G, Liu CC (2022). ApoE in Alzheimer’s disease: pathophysiology and therapeutic strategies. Mol Neurodegener.

[148] Martens YA, Zhao N, Liu CC, Kanekiyo T, Yang AJ, Goate AM (2022). ApoE Cascade Hypothesis in the pathogenesis of Alzheimer’s disease and related dementias. Neuron.

[149] Koutsodendris N, Nelson MR, Rao A, Huang Y (2022). Apolipoprotein E and Alzheimer’s Disease: findings, hypotheses, and potential mechanisms. Annu Rev Pathol.

[150] Lautner R, Palmqvist S, Mattsson N, Andreasson U, Wallin A, Palsson E (2014). Apolipoprotein E genotype and the diagnostic accuracy of cerebrospinal fluid biomarkers for Alzheimer disease. JAMA Psychiatry.

[151] Shi Y, Yamada K, Liddelow SA, Smith ST, Zhao L, Luo W (2017). ApoE4 markedly exacerbates tau-mediated neurodegeneration in a mouse model of tauopathy. Nature.

[152] Davis AA, Inman CE, Wargel ZM, Dube U, Freeberg BM, Galluppi A et al. APOE genotype regulates pathology and disease progression in synucleinopathy. Sci Transl Med. 2020;12(529). 10.1126/scitranslmed.aay3069 PMC7289511 32024799

[153] Tran TTT, Corsini S, Kellingray L, Hegarty C, Le Gall G, Narbad A (2019). APOE genotype influences the gut microbiome structure and function in humans and mice: relevance for Alzheimer’s disease pathophysiology. FASEB J.

[154] Nunes AF, Amaral JD, Lo AC, Fonseca MB, Viana RJ, Callaerts-Vegh Z (2012). TUDCA, a bile acid, attenuates amyloid precursor protein processing and amyloid-beta deposition in APP/PS1 mice. Mol Neurobiol.

[155] Yeh FL, Wang Y, Tom I, Gonzalez LC, Sheng M (2016). TREM2 binds to Apolipoproteins, including APOE and CLU/APOJ, and thereby facilitates uptake of amyloid-Beta by Microglia. Neuron.

[156] Filipello F, Morini R, Corradini I, Zerbi V, Canzi A, Michalski B (2018). The Microglial Innate Immune receptor TREM2 is required for synapse elimination and normal brain connectivity. Immunity.

[157] Zhou Y, Song WM, Andhey PS, Swain A, Levy T, Miller KR (2020). Human and mouse single-nucleus transcriptomics reveal TREM2-dependent and TREM2-independent cellular responses in Alzheimer’s disease. Nat Med.

[158] Ulland TK, Song WM, Huang SC, Ulrich JD, Sergushichev A, Beatty WL (2017). TREM2 maintains microglial metabolic fitness in Alzheimer’s Disease. Cell.

[159] Nugent AA, Lin K, van Lengerich B, Lianoglou S, Przybyla L, Davis SS (2020). TREM2 regulates microglial cholesterol metabolism upon chronic phagocytic challenge. Neuron.

[160] Deczkowska A, Weiner A, Amit I (2020). The Physiology, Pathology, and potential therapeutic applications of the TREM2 signaling pathway. Cell.

[161] Zhao J, Bi W, Xiao S, Lan X, Cheng X, Zhang J (2019). Neuroinflammation induced by lipopolysaccharide causes cognitive impairment in mice. Sci Rep.

[162] Wang Y, Lin Y, Wang L, Zhan H, Luo X, Zeng Y (2020). TREM2 ameliorates neuroinflammatory response and cognitive impairment via PI3K/AKT/FoxO3a signaling pathway in Alzheimer’s disease mice. Aging.

[163] Li H, Liu F, Jiang W, Wang K, Cao X, Zou J (2022). TREM2 ameliorates Lipopolysaccharide-Induced oxidative stress response and neuroinflammation by promoting Sirtuin3 in BV2 cells. Neurotox Res.

[164] Li R, Zhang J, Wang Q, Cheng M, Lin B (2022). TPM1 mediates inflammation downstream of TREM2 via the PKA/CREB signaling pathway. J Neuroinflammation.

[165] Lewandowski CT, Laham MS, Thatcher GRJ (2022). Remembering your A, B, C’s: Alzheimer’s disease and ABCA1. Acta Pharm Sin B.

[166] Nordestgaard LT, Tybjaerg-Hansen A, Nordestgaard BG, Frikke-Schmidt R (2015). Loss-of-function mutation in ABCA1 and risk of Alzheimer’s disease and cerebrovascular disease. Alzheimers Dement.

[167] Wahrle SE, Jiang H, Parsadanian M, Kim J, Li A, Knoten A (2008). Overexpression of ABCA1 reduces amyloid deposition in the PDAPP mouse model of Alzheimer disease. J Clin Invest.

[168] Holstege H, Hulsman M, Charbonnier C, Grenier-Boley B, Quenez O, Grozeva D (2022). Exome sequencing identifies rare damaging variants in ATP8B4 and ABCA1 as risk factors for Alzheimer’s disease. Nat Genet.

[169] Du Y, Li X, Su C, Xi M, Zhang X, Jiang Z (2020). Butyrate protects against high-fat diet-induced atherosclerosis via up-regulating ABCA1 expression in apolipoprotein E-deficiency mice. Br J Pharmacol.

[170] Mohammadi A, Najar AG, Yaghoobi MM, Jahani Y, Vahabzadeh Z (2016). Trimethylamine-N-Oxide treatment induces changes in the ATP-Binding Cassette Transporter A1 and scavenger receptor A1 in murine macrophage J774A.1 cells. Inflammation.

[171] Yang Y, Karampoor S, Mirzaei R, Borozdkin L, Zhu P (2023). The interplay between microbial metabolites and macrophages in cardiovascular diseases: a comprehensive review. Int Immunopharmacol.

[172] Moulton MJ, Barish S, Ralhan I, Chang J, Goodman LD, Harland JG (2021). Neuronal ROS-induced glial lipid droplet formation is altered by loss of Alzheimer’s disease-associated genes. Proc Natl Acad Sci U S A.

[173] Steinberg S, Stefansson H, Jonsson T, Johannsdottir H, Ingason A, Helgason H (2015). Loss-of-function variants in ABCA7 confer risk of Alzheimer’s disease. Nat Genet.

[174] Satoh K, Abe-Dohmae S, Yokoyama S, St George-Hyslop P, Fraser PE (2015). ATP-binding cassette transporter A7 (ABCA7) loss of function alters Alzheimer amyloid processing. J Biol Chem.

[175] Aikawa T, Ren Y, Holm ML, Asmann YW, Alam A, Fitzgerald ML (2021). ABCA7 regulates brain fatty acid metabolism during LPS-Induced Acute inflammation. Front Neurosci.

[176] Wojtas AM, Kang SS, Olley BM, Gatherer M, Shinohara M, Lozano PA (2017). Loss of clusterin shifts amyloid deposition to the cerebrovasculature via disruption of perivascular drainage pathways. Proc Natl Acad Sci U S A.

[177] Jun YK, Yoon HT, Kwon SH, Jo UH, Kim JE, Han YM (2023). Regulation of psoriasis, colitis, and the intestinal microbiota by clusterin. Sci Rep.

[178] Wang QJ, Shen YE, Wang X, Fu S, Zhang X, Zhang YN (2020). Concomitant memantine and Lactobacillus plantarum treatment attenuates cognitive impairments in APP/PS1 mice. Aging.

[179] Brown MS, Goldstein JL (1997). The SREBP pathway: regulation of cholesterol metabolism by proteolysis of a membrane-bound transcription factor. Cell.

[180] Shimano H, Sato R (2017). SREBP-regulated lipid metabolism: convergent physiology - divergent pathophysiology. Nat Rev Endocrinol.

[181] Shah SA, Yoon GH, Chung SS, Abid MN, Kim TH, Lee HY (2017). Osmotin reduced amyloid beta (abeta) burden by inhibiting SREBP2 expression in APP/PS1 mice. Mol Psychiatry.

[182] Shah SA, Yoon GH, Chung SS, Abid MN, Kim TH, Lee HY (2017). Novel osmotin inhibits SREBP2 via the AdipoR1/AMPK/SIRT1 pathway to improve Alzheimer’s disease neuropathological deficits. Mol Psychiatry.

[183] Vourakis M, Mayer G, Rousseau G. The role of gut microbiota on cholesterol metabolism in atherosclerosis. Int J Mol Sci. 2021;22(15). 10.3390/ijms22158074 PMC8347163 34360839

[184] Jucker M, Walker LC (2023). Alzheimer’s disease: from immunotherapy to immunoprevention. Cell.

[185] Zhang Y, Chen H, Li R, Sterling K, Song W (2023). Amyloid beta-based therapy for Alzheimer’s disease: challenges, successes and future. Signal Transduct Target Ther.

[186] Campos-Pena V, Pichardo-Rojas P, Sanchez-Barbosa T, Ortiz-Islas E, Rodriguez-Perez CE, Montes P et al. Amyloid beta, Lipid Metabolism, Basal Cholinergic System, and Therapeutics in Alzheimer’s Disease. Int J Mol Sci. 2022;23(20). 10.3390/ijms232012092 PMC9603563 36292947

[187] Moll T, Marshall JNG, Soni N, Zhang S, Cooper-Knock J, Shaw PJ (2021). Membrane lipid raft homeostasis is directly linked to neurodegeneration. Essays Biochem.

[188] Bode DC, Freeley M, Nield J, Palma M, Viles JH (2019). Amyloid-beta oligomers have a profound detergent-like effect on lipid membrane bilayers, imaged by atomic force and electron microscopy. J Biol Chem.

[189] Kiriyama Y, Nochi H. The biosynthesis, signaling, and neurological functions of bile acids. Biomolecules. 2019;9(6). 10.3390/biom9060232 PMC6628048 31208099

[190] Liu S, Gao J, Zhu M, Liu K, Zhang HL (2020). Gut microbiota and Dysbiosis in Alzheimer’s Disease: implications for Pathogenesis and treatment. Mol Neurobiol.

[191] Drummond E, Pires G, MacMurray C, Askenazi M, Nayak S, Bourdon M (2020). Phosphorylated tau interactome in the human Alzheimer’s disease brain. Brain.

[192] Guo T, Zhang D, Zeng Y, Huang TY, Xu H, Zhao Y (2020). Molecular and cellular mechanisms underlying the pathogenesis of Alzheimer’s disease. Mol Neurodegener.

[193] Wang H, Kulas JA, Wang C, Holtzman DM, Ferris HA, Hansen SB (2021). Regulation of beta-amyloid production in neurons by astrocyte-derived cholesterol. Proc Natl Acad Sci U S A.

[194] Xia Y, Xiao Y, Wang ZH, Liu X, Alam AM, Haran JP (2023). Bacteroides Fragilis in the gut microbiomes of Alzheimer’s disease activates microglia and triggers pathogenesis in neuronal C/EBPbeta transgenic mice. Nat Commun.

[195] Song X, Zhao Z, Zhao Y, Wang Z, Wang C, Yang G (2022). Lactobacillus plantarum DP189 prevents cognitive dysfunction in D-galactose/AlCl(3) induced mouse model of Alzheimer’s disease via modulating gut microbiota and PI3K/Akt/GSK-3beta signaling pathway. Nutr Neurosci.

[196] Qian XH, Song XX, Liu XL, Chen SD, Tang HD (2021). Inflammatory pathways in Alzheimer’s disease mediated by gut microbiota. Ageing Res Rev.

[197] Heneka MT, Carson MJ, El Khoury J, Landreth GE, Brosseron F, Feinstein DL (2015). Neuroinflammation in Alzheimer’s disease. Lancet Neurol.

[198] Akiyama H, Barger S, Barnum S, Bradt B, Bauer J, Cole GM (2000). Inflammation and Alzheimer’s disease. Neurobiol Aging.

[199] Bairamian D, Sha S, Rolhion N, Sokol H, Dorothee G, Lemere CA (2022). Microbiota in neuroinflammation and synaptic dysfunction: a focus on Alzheimer’s disease. Mol Neurodegener.

[200] Haran JP, Bhattarai SK, Foley SE, Dutta P, Ward DV, Bucci V et al. Alzheimer’s Disease Microbiome Is Associated with Dysregulation of the Anti-Inflammatory P-Glycoprotein Pathway. mBio. 2019;10(3). 10.1128/mBio.00632-19 PMC6509190 31064831

[201] Erny D, Hrabe de Angelis AL, Jaitin D, Wieghofer P, Staszewski O, David E (2015). Host microbiota constantly control maturation and function of microglia in the CNS. Nat Neurosci.

[202] Rothhammer V, Mascanfroni ID, Bunse L, Takenaka MC, Kenison JE, Mayo L (2016). Type I interferons and microbial metabolites of tryptophan modulate astrocyte activity and central nervous system inflammation via the aryl hydrocarbon receptor. Nat Med.

[203] Dodiya HB, Lutz HL, Weigle IQ, Patel P, Michalkiewicz J, Roman-Santiago CJ et al. Gut microbiota-driven brain abeta amyloidosis in mice requires microglia. J Exp Med. 2022;219(1). 10.1084/jem.20200895 PMC8647415 34854884

[204] Chandra S, Di Meco A, Dodiya HB, Popovic J, Cuddy LK, Weigle IQ (2023). The gut microbiome regulates astrocyte reaction to Abeta amyloidosis through microglial dependent and independent mechanisms. Mol Neurodegener.

[205] Minter MR, Hinterleitner R, Meisel M, Zhang C, Leone V, Zhang X (2017). Antibiotic-induced perturbations in microbial diversity during post-natal development alters amyloid pathology in an aged APP(SWE)/PS1(DeltaE9) murine model of Alzheimer’s disease. Sci Rep.

[206] Chen C, Ahn EH, Kang SS, Liu X, Alam A, Ye K (2020). Gut dysbiosis contributes to amyloid pathology, associated with C/EBPbeta/AEP signaling activation in Alzheimer’s disease mouse model. Sci Adv.

[207] Sies H, Jones DP (2020). Reactive oxygen species (ROS) as pleiotropic physiological signalling agents. Nat Rev Mol Cell Biol.

[208] Shandilya S, Kumar S, Kumar Jha N, Kumar Kesari K, Ruokolainen J (2022). Interplay of gut microbiota and oxidative stress: perspective on neurodegeneration and neuroprotection. J Adv Res.

[209] Schieber M, Chandel NS (2014). ROS function in redox signaling and oxidative stress. Curr Biol.

[210] Fao L, Mota SI, Rego AC (2019). Shaping the Nrf2-ARE-related pathways in Alzheimer’s and Parkinson’s diseases. Ageing Res Rev.

[211] Szentirmai E, Millican NS, Massie AR, Kapas L (2019). Butyrate, a metabolite of intestinal bacteria, enhances sleep. Sci Rep.

[212] Doifode T, Giridharan VV, Generoso JS, Bhatti G, Collodel A, Schulz PE (2021). The impact of the microbiota-gut-brain axis on Alzheimer’s disease pathophysiology. Pharmacol Res.

[213] Chambers ES, Preston T, Frost G, Morrison DJ (2018). Role of gut microbiota-generated short-chain fatty acids in metabolic and Cardiovascular Health. Curr Nutr Rep.

[214] Sivandzade F, Prasad S, Bhalerao A, Cucullo L (2019). NRF2 and NF-қB interplay in cerebrovascular and neurodegenerative disorders: molecular mechanisms and possible therapeutic approaches. Redox Biol.

[215] Seo EJ, Fischer N, Efferth T (2018). Phytochemicals as inhibitors of NF-kappaB for treatment of Alzheimer’s disease. Pharmacol Res.

[216] Wang L, Zhang X, Xiong X, Zhu H, Chen R, Zhang S et al. Nrf2 regulates oxidative stress and its role in cerebral ischemic stroke. Antioxid (Basel). 2022;11(12). 10.3390/antiox11122377 PMC9774301 36552584

[217] Kerr JS, Adriaanse BA, Greig NH, Mattson MP, Cader MZ, Bohr VA (2017). Mitophagy and Alzheimer’s Disease: Cellular and Molecular mechanisms. Trends Neurosci.

[218] Shoshan-Barmatz V, Nahon-Crystal E, Shteinfer-Kuzmine A, Gupta R (2018). VDAC1, mitochondrial dysfunction, and Alzheimer’s disease. Pharmacol Res.

[219] Yoo W, Zieba JK, Foegeding NJ, Torres TP, Shelton CD, Shealy NG (2021). High-fat diet-induced colonocyte dysfunction escalates microbiota-derived trimethylamine N-oxide. Science.

[220] Mottawea W, Chiang CK, Muhlbauer M, Starr AE, Butcher J, Abujamel T (2016). Altered intestinal microbiota-host mitochondria crosstalk in new onset Crohn’s disease. Nat Commun.

[221] Mossad O, Batut B, Yilmaz B, Dokalis N, Mezo C, Nent E (2022). Gut microbiota drives age-related oxidative stress and mitochondrial damage in microglia via the metabolite N(6)-carboxymethyllysine. Nat Neurosci.

[222] Wang C, Zheng D, Weng F, Jin Y, He L (2022). Sodium butyrate ameliorates the cognitive impairment of Alzheimer’s disease by regulating the metabolism of astrocytes. Psychopharmacology.

[223] Sharma VK, Mehta V, Singh TG (2020). Alzheimer’s disorder: epigenetic connection and Associated Risk factors. Curr Neuropharmacol.

[224] Nativio R, Lan Y, Donahue G, Sidoli S, Berson A, Srinivasan AR (2020). An integrated multi-omics approach identifies epigenetic alterations associated with Alzheimer’s disease. Nat Genet.

[225] Peleg S, Sananbenesi F, Zovoilis A, Burkhardt S, Bahari-Javan S, Agis-Balboa RC (2010). Altered histone acetylation is associated with age-dependent memory impairment in mice. Science.

[226] Graff J, Rei D, Guan JS, Wang WY, Seo J, Hennig KM (2012). An epigenetic blockade of cognitive functions in the neurodegenerating brain. Nature.

[227] Guan JS, Haggarty SJ, Giacometti E, Dannenberg JH, Joseph N, Gao J (2009). HDAC2 negatively regulates memory formation and synaptic plasticity. Nature.

[228] Lin Y, Lin A, Cai L, Huang W, Yan S, Wei Y (2023). ACSS2-dependent histone acetylation improves cognition in mouse model of Alzheimer’s disease. Mol Neurodegener.

[229] Kesika P, Suganthy N, Sivamaruthi BS, Chaiyasut C (2021). Role of gut-brain axis, gut microbial composition, and probiotic intervention in Alzheimer’s disease. Life Sci.

[230] Bonfili L, Cecarini V, Gogoi O, Berardi S, Scarpona S, Angeletti M (2020). Gut microbiota manipulation through probiotics oral administration restores glucose homeostasis in a mouse model of Alzheimer’s disease. Neurobiol Aging.

[231] Bonfili L, Cecarini V, Berardi S, Scarpona S, Suchodolski JS, Nasuti C (2017). Microbiota modulation counteracts Alzheimer’s disease progression influencing neuronal proteolysis and gut hormones plasma levels. Sci Rep.

[232] Bonfili L, Cecarini V, Cuccioloni M, Angeletti M, Berardi S, Scarpona S (2018). SLAB51 probiotic formulation activates SIRT1 pathway promoting antioxidant and neuroprotective effects in an AD mouse model. Mol Neurobiol.

[233] Menden A, Hall D, Hahn-Townsend C, Broedlow CA, Joshi U, Pearson A (2022). Exogenous lipase administration alters gut microbiota composition and ameliorates Alzheimer’s disease-like pathology in APP/PS1 mice. Sci Rep.

[234] Kaur H, Nagamoto-Combs K, Golovko S, Golovko MY, Klug MG, Combs CK (2020). Probiotics ameliorate intestinal pathophysiology in a mouse model of Alzheimer’s disease. Neurobiol Aging.

[235] Abraham D, Feher J, Scuderi GL, Szabo D, Dobolyi A, Cservenak M (2019). Exercise and probiotics attenuate the development of Alzheimer’s disease in transgenic mice: role of microbiome. Exp Gerontol.

[236] Shamsipour S, Sharifi G, Taghian F (2021). An 8-Week Administration of Bifidobacterium bifidum and Lactobacillus plantarum combined with Exercise Training alleviates neurotoxicity of abeta and spatial learning via Acetylcholine in Alzheimer Rat Model. J Mol Neurosci.

[237] Sorboni SG, Moghaddam HS, Jafarzadeh-Esfehani R, Soleimanpour S (2022). A Comprehensive Review on the role of the gut Microbiome in Human Neurological disorders. Clin Microbiol Rev.

[238] Zhang S, Lv S, Li Y, Wei D, Zhou X, Niu X (2023). Prebiotics modulate the microbiota-gut-brain axis and ameliorate cognitive impairment in APP/PS1 mice. Eur J Nutr.

[239] Lee YS, Lai DM, Huang HJ, Lee-Chen GJ, Chang CH, Hsieh-Li HM (2021). Prebiotic lactulose ameliorates the cognitive deficit in Alzheimer’s Disease Mouse Model through Macroautophagy and chaperone-mediated autophagy pathways. J Agric Food Chem.

[240] Liu Q, Xi Y, Wang Q, Liu J, Li P, Meng X (2021). Mannan oligosaccharide attenuates cognitive and behavioral disorders in the 5xFAD Alzheimer’s disease mouse model via regulating the gut microbiota-brain axis. Brain Behav Immun.

[241] Sun J, Xu J, Ling Y, Wang F, Gong T, Yang C (2019). Fecal microbiota transplantation alleviated Alzheimer’s disease-like pathogenesis in APP/PS1 transgenic mice. Transl Psychiatry.

[242] Kim N, Jeon SH, Ju IG, Gee MS, Do J, Oh MS (2021). Transplantation of gut microbiota derived from Alzheimer’s disease mouse model impairs memory function and neurogenesis in C57BL/6 mice. Brain Behav Immun.

[243] Jin J, Xu Z, Zhang L, Zhang C, Zhao X, Mao Y (2023). Gut-derived beta-amyloid: likely a centerpiece of the gut-brain axis contributing to Alzheimer’s pathogenesis. Gut Microbes.

[244] Kobayashi Y, Sugahara H, Shimada K, Mitsuyama E, Kuhara T, Yasuoka A (2017). Therapeutic potential of Bifidobacterium breve strain A1 for preventing cognitive impairment in Alzheimer’s disease. Sci Rep.

[245] Lee HJ, Lee KE, Kim JK, Kim DH (2019). Suppression of gut dysbiosis by Bifidobacterium longum alleviates cognitive decline in 5XFAD transgenic and aged mice. Sci Rep.

[246] Wu Y, Niu X, Li P, Tong T, Wang Q, Zhang M (2023). Lactobacillaceae improve cognitive dysfunction via regulating gut microbiota and suppressing Abeta deposits and neuroinflammation in APP/PS1 mice. Arch Microbiol.

[247] Cao J, Amakye WK, Qi C, Liu X, Ma J, Ren J (2021). Bifidobacterium Lactis Probio-M8 regulates gut microbiota to alleviate Alzheimer’s disease in the APP/PS1 mouse model. Eur J Nutr.

[248] Sun J, Xu J, Yang B, Chen K, Kong Y, Fang N (2020). Effect of Clostridium butyricum against microglia-mediated neuroinflammation in Alzheimer’s Disease via regulating gut microbiota and metabolites Butyrate. Mol Nutr Food Res.

[249] Xin Y, Diling C, Jian Y, Ting L, Guoyan H, Hualun L (2018). Effects of oligosaccharides from Morinda Officinalis on Gut Microbiota and Metabolome of APP/PS1 transgenic mice. Front Neurol.

[250] Shabbir U, Tyagi A, Elahi F, Aloo SO, Oh DH. The Potential Role of Polyphenols in Oxidative Stress and Inflammation Induced by Gut Microbiota in Alzheimer’s Disease. Antioxid (Basel). 2021;10(9). 10.3390/antiox10091370 PMC8472599 34573002

[251] Zhang J, Hao J, Liu R, Wu T, Liu R, Sui W (2022). Hawthorn flavonoid ameliorates cognitive deficit in mice with Alzheimer’s disease by increasing the levels of Bifidobacteriales in gut microbiota and docosapentaenoic acid in serum metabolites. Food Funct.

[252] Sun ZZ, Li XY, Wang S, Shen L, Ji HF (2020). Bidirectional interactions between curcumin and gut microbiota in transgenic mice with Alzheimer’s disease. Appl Microbiol Biotechnol.

[253] Zhang J, Zheng Y, Luo Y, Du Y, Zhang X, Fu J (2019). Curcumin inhibits LPS-induced neuroinflammation by promoting microglial M2 polarization via TREM2/ TLR4/ NF-kappaB pathways in BV2 cells. Mol Immunol.

[254] Li J, Zhao R, Jiang Y, Xu Y, Zhao H, Lyu X (2020). Bilberry anthocyanins improve neuroinflammation and cognitive dysfunction in APP/PSEN1 mice via the CD33/TREM2/TYROBP signaling pathway in microglia. Food Funct.

[255] Xu J, Chen HB, Li SL (2017). Understanding the Molecular mechanisms of the interplay between Herbal Medicines and Gut Microbiota. Med Res Rev.

[256] Xu QQ, Su ZR, Yang W, Zhong M, Xian YF, Lin ZX (2023). Patchouli alcohol attenuates the cognitive deficits in a transgenic mouse model of Alzheimer’s disease via modulating neuropathology and gut microbiota through suppressing C/EBPbeta/AEP pathway. J Neuroinflammation.

[257] Fu J, Li J, Sun Y, Liu S, Song F, Liu Z (2023). In-depth investigation of the mechanisms of Schisandra chinensis polysaccharide mitigating Alzheimer’s disease rat via gut microbiota and feces metabolomics. Int J Biol Macromol.

[258] Wang Y, Wang M, Fan K, Li T, Yan T, Wu B (2018). Protective effects of Alpinae Oxyphyllae Fructus extracts on lipopolysaccharide-induced animal model of Alzheimer’s disease. J Ethnopharmacol.

[259] Xu M, Yang Y, Peng J, Zhang Y, Wu B, He B et al. Effects of Alpinae Oxyphyllae Fructus on microglial polarization in a LPS-induced BV2 cells model of neuroinflammation via TREM2. J Ethnopharmacol. 2023;302(Pt A):115914. 10.1016/j.jep.2022.115914 36347303

[260] Shi K, Chen L, Chen L, Tan A, Xie G, Long Q (2022). Epimedii Folium and Curculiginis Rhizoma ameliorate lipopolysaccharides-induced cognitive impairment by regulating the TREM2 signaling pathway. J Ethnopharmacol.

[261] Sun Y, Zhang H, Liu R, Huang R, Zhang X, Zhou S (2024). Pyrolae herba alleviates cognitive impairment via hippocampal TREM2 signaling modulating neuroinflammation and neurogenesis in lipopolysaccharide-treated mice. J Ethnopharmacol.

[262] Kim J, Lee H, An J, Song Y, Lee CK, Kim K (2019). Alterations in gut microbiota by statin therapy and possible Intermediate effects on Hyperglycemia and Hyperlipidemia. Front Microbiol.

[263] Zahedi E, Sanaeierad A, Nikbakhtzadeh M, Roghani M, Zamani E (2023). Simvastatin improves learning and memory impairment via gut-brain axis regulation in an ovariectomized/D-galactose Alzheimer’s rat model. Behav Brain Res.

[264] Daily JW, Kang S, Park S (2021). Protection against Alzheimer’s disease by luteolin: role of brain glucose regulation, anti-inflammatory activity, and the gut microbiota-liver-brain axis. BioFactors.

[265] Xu TC, Lv Y, Liu QY, Chen HS (2022). Long-term atorvastatin improves cognitive decline by regulating gut function in naturally ageing rats. Immun Ageing.

[266] Pellegrini C, Antonioli L, Calderone V, Colucci R, Fornai M, Blandizzi C (2020). Microbiota-gut-brain axis in health and disease: is NLRP3 inflammasome at the crossroads of microbiota-gut-brain communications?. Prog Neurobiol.

[267] Nagpal R, Neth BJ, Wang S, Craft S, Yadav H (2019). Modified Mediterranean-ketogenic diet modulates gut microbiome and short-chain fatty acids in association with Alzheimer’s disease markers in subjects with mild cognitive impairment. EBioMedicine.

[268] Ma D, Wang AC, Parikh I, Green SJ, Hoffman JD, Chlipala G (2018). Ketogenic diet enhances neurovascular function with altered gut microbiome in young healthy mice. Sci Rep.

[269] Wegierska AE, Charitos IA, Topi S, Potenza MA, Montagnani M, Santacroce L (2022). The connection between Physical Exercise and Gut Microbiota: implications for competitive sports athletes. Sports Med.

[270] Dohnalova L, Lundgren P, Carty JRE, Goldstein N, Wenski SL, Nanudorn P (2022). A microbiome-dependent gut-brain pathway regulates motivation for exercise. Nature.

[271] Dalton A, Mermier C, Zuhl M (2019). Exercise influence on the microbiome-gut-brain axis. Gut Microbes.

[272] Du Z, Li Y, Li J, Zhou C, Li F, Yang X (2018). Physical activity can improve cognition in patients with Alzheimer’s disease: a systematic review and meta-analysis of randomized controlled trials. Clin Interv Aging.

[273] Gubert C, Kong G, Renoir T, Hannan AJ (2020). Exercise, diet and stress as modulators of gut microbiota: implications for neurodegenerative diseases. Neurobiol Dis.

[274] Kang SS, Jeraldo PR, Kurti A, Miller ME, Cook MD, Whitlock K (2014). Diet and exercise orthogonally alter the gut microbiome and reveal independent associations with anxiety and cognition. Mol Neurodegener.

[275] Yuan S, Yang J, Jian Y, Lei Y, Yao S, Hu Z (2022). Treadmill Exercise modulates intestinal microbes and suppresses LPS displacement to Alleviate Neuroinflammation in the brains of APP/PS1 mice. Nutrients.

[276] Zhang Y, Wang G, Li R, Liu R, Yu Z, Zhang Z (2023). Trimethylamine N-oxide aggravated cognitive impairment from APP/PS1 mice and protective roles of voluntary exercise. Neurochem Int.

