# Supplementary material for: Gut microbiota-host lipid crosstalk in Alzheimer’s disease: implications for disease progression and therapeutics
Source: Mol Neurodegener. 2024 Apr 16;19:35. doi: 10.1186/s13024-024-00720-0 (PMC11020986; doi:10.1186/s13024-024-00720-0)
Supplement: Supplementary file 1 — Supplementary Material 1 [file 13024_2024_720_MOESM1_ESM.pdf]

This supplementary document is a publication license for our illustrations.

Illustration Publication License

The illustrations of our manuscript have been created using the BioRender.com. To ensure compliance with publishing regulations, we have obtained Publication License from Biorender specifically for the purpose of academic paper publication.

Figure 1

49 Spadina Ave. Suite 200
Toronto ON M5V 2J1 Canada
www.biorender.com

Confirmation of Publication and Licensing Rights

February 29th, 2024
Science Suite Inc.

Subscription:	*Postdoc Plan*
Agreement number:	*TV26IPOOLQ*
Journal name:	*Molecular Neurodegeneration*

To whom this may concern,

This document is to confirm that Yaxi Luo has been granted a license to use the BioRender content, including icons, templates and other original artwork, appearing in the attached completed graphic pursuant to BioRender's Academic License Terms. This license permits BioRender content to be sublicensed for use in journal publications.

All rights and ownership of BioRender content are reserved by BioRender. All completed graphics must be accompanied by the following citation: "Created with BioRender.com".

BioRender content included in the completed graphic is not licensed for any commercial uses beyond publication in a journal. For any commercial use of this figure, users may, if allowed, recreate it in BioRender under an Industry BioRender Plan.

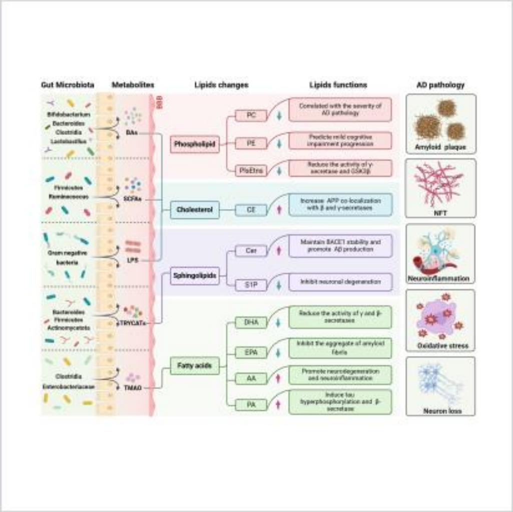

For any questions regarding this document, or other questions about publishing with BioRender refer to our BioRender Publication Guide, or contact BioRender Support at support@biorender.com.

Figure 2

bio RENDER

49 Spadina Ave. Suite 200
Toronto ON M5V 2J1 Canada
www.biorender.com

Confirmation of Publication and Licensing Rights

February 29th, 2024
Science Suite Inc.

Subscription:	*Postdoc Plan*
Agreement number:	*FR26IPO7DW*
Journal name:	*Molecular Neurodegeneration*

To whom this may concern,

This document is to confirm that Yaxi Luo has been granted a license to use the BioRender content, including icons, templates and other original artwork, appearing in the attached completed graphic pursuant to BioRender's Academic License Terms. This license permits BioRender content to be sublicensed for use in journal publications.

All rights and ownership of BioRender content are reserved by BioRender. All completed graphics must be accompanied by the following citation: “Created with BioRender.com”.

BioRender content included in the completed graphic is not licensed for any commercial uses beyond publication in a journal. For any commercial use of this figure, users may, if allowed, recreate it in BioRender under an Industry BioRender Plan.

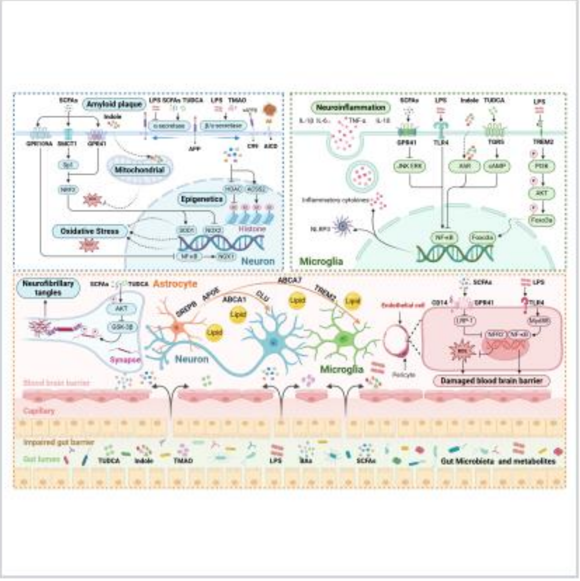

For any questions regarding this document, or other questions about publishing with BioRender refer to our BioRender Publication Guide, or contact BioRender Support at support@biorender.com.

Figure 3

bio RENDER

49 Spadina Ave. Suite 200
Toronto ON M5V 2J1 Canada
www.biorender.com

Confirmation of Publication and Licensing Rights

February 29th, 2024
Science Suite Inc.

Subscription:	*Postdoc Plan*
Agreement number:	*IR26IPPD3Q*
Journal name:	*Molecular Neurodegeneration*

To whom this may concern,

This document is to confirm that Yaxi Luo has been granted a license to use the BioRender content, including icons, templates and other original artwork, appearing in the attached completed graphic pursuant to BioRender's Academic License Terms. This license permits BioRender content to be sublicensed for use in journal publications.

All rights and ownership of BioRender content are reserved by BioRender. All completed graphics must be accompanied by the following citation: "Created with BioRender.com".

BioRender content included in the completed graphic is not licensed for any commercial uses beyond publication in a journal. For any commercial use of this figure, users may, if allowed, recreate it in BioRender under an Industry BioRender Plan.

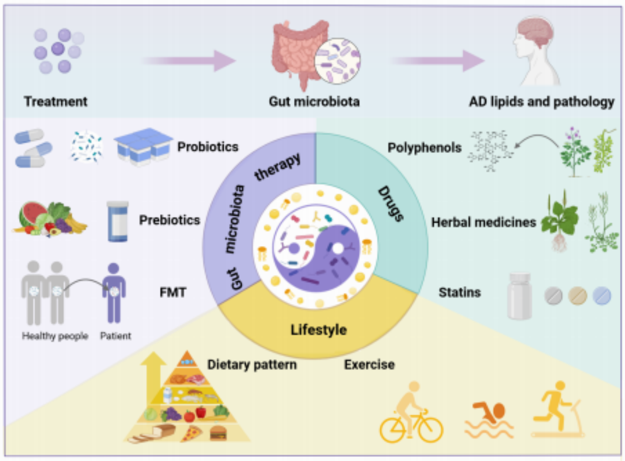

For any questions regarding this document, or other questions about publishing with BioRender refer to our BioRender Publication Guide, or contact BioRender Support at support@biorender.com.
